# Identification of a Latin American-specific BabA adhesin variant through whole genome sequencing of *Helicobacter pylori* patient isolates from Nicaragua

**DOI:** 10.1186/s12862-016-0619-y

**Published:** 2016-02-29

**Authors:** Kaisa Thorell, Shaghayegh Hosseini, Reyna Victoria Palacios Palacios Gonzáles, Chatchai Chaotham, David Y. Graham, Lawrence Paszat, Linda Rabeneck, Samuel B. Lundin, Intawat Nookaew, Åsa Sjöling

**Affiliations:** Department of Microbiology and Immunology, Sahlgrenska Academy, University of Gothenburg, Gothenburg, Sweden; Department of Biology and Biological Engineering, Chalmers University of Technology, Gothenburg, Sweden; Laboratorio de Patología, Hospital Salud Integral, Managua, Nicaragua; Department of Medicine, Michael E. DeBakey VA Medical Center and Baylor College of Medicine, Houston, TX USA; Dalla Lana School of Public Health, University of Toronto, Toronto, Canada; Cancer Care Ontario, University of Toronto, Toronto, Canada; Present address: Comparative Genomics Group, Biosciences Division, Oak Ridge National Laboratory, Oak Ridge, TN USA; Present address: Department of Microbiology, Tumor and Cell Biology, Karolinska Institutet, Box 280, 171 77 Stockholm, Sweden

**Keywords:** Helicobacter, Whole-genome sequencing, Phylogeny, Virulence factors, BabA

## Abstract

**Background:**

*Helicobacter pylori (H. pylori)* is one of the most common bacterial infections in humans and this infection can lead to gastric ulcers and gastric cancer. *H. pylori* is one of the most genetically variable human pathogens and the ability of the bacterium to bind to the host epithelium as well as the presence of different virulence factors and genetic variants within these genes have been associated with disease severity. Nicaragua has particularly high gastric cancer incidence and we therefore studied Nicaraguan clinical *H. pylori* isolates for factors that could contribute to cancer risk.

**Methods:**

The complete genomes of fifty-two Nicaraguan *H. pylori * isolates were sequenced and assembled *de novo,* and phylogenetic and virulence factor analyses were performed.

**Results:**

The Nicaraguan isolates showed phylogenetic relationship with West African isolates in whole-genome sequence comparisons and with Western and urban South- and Central American isolates using MLSA (Multi-locus sequence analysis). A majority, 77 % of the isolates carried the cancer-associated virulence gene *cagA* and also the s1/i1/m1 vacuolating cytotoxin, *vacA* allele combination, which is linked to increased severity of disease. Specifically, we also found that Nicaraguan isolates have a blood group-binding adhesin (BabA) variant highly similar to previously reported BabA sequences from Latin America, including from isolates belonging to other phylogenetic groups. These BabA sequences were found to be under positive selection at several amino acid positions that differed from the global collection of isolates.

**Conclusion:**

The discovery of a Latin American BabA variant, independent of overall phylogenetic background, suggests hitherto unknown host or environmental factors within the Latin American population giving *H. pylori* isolates carrying this adhesin variant a selective advantage, which could affect pathogenesis and risk for sequelae through specific adherence properties.

**Electronic supplementary material:**

The online version of this article (doi:10.1186/s12862-016-0619-y) contains supplementary material, which is available to authorized users.

## Background

*Helicobacter pylori* (*H. pylori)* is a Gram-negative, spiral-shaped bacterium that resides in the stomach of about half the world’s population. Infection with *H. pylori* causes progressive, acute and chronic inflammation (gastritis) that remains undetected in a majority of infected individuals. However, *H. pylori* infection is able to cause severe clinical outcomes such as duodenal and gastric ulcers, and is classified as a carcinogen causing gastric adenocarcinoma and mucosa-associated lymphoid tissue (MALT) lymphoma. These more severe clinical outcomes present as ulcers in approximately 10-15 % of all infected individuals, and as gastric cancer in an additional 1-3 % [1]. The incidence rates of these diseases vary world-wide with e.g., considerably higher incidence of gastric cancer in East Asia, Central America and South America [2]. What leads to this divergence in clinical outcome is not entirely known, but both host genetics modulating the immune response towards the infection, as well as bacterial genetics and environmental factors such as smoking and high intake of salt has been shown to play a role [3].

*H. pylori* has one of the highest mutation and recombination rates observed in pathogenic bacteria [4, 5] with a much higher recombination frequency than frequency of point mutations [6]. Hence, *H. pylori* show extensive intra species diversity but it also has a clearly traceable phylogeny reflecting the ancestry of the carrier and the migration of ancient human groups [7, 8]. This has been investigated thoroughly, especially using multi-locus sequence typing (MLST) but also, in recent years, based on the growing number of whole-genome sequenced isolates [9, 10]. Six major geographical groups with different characteristics have been identified for *H. pylori,* reflecting the migratory waves of human populations throughout history. These groups are hpEurope, hpAsia2, hpAfrica2, hpNEAfrica, and hpSahul, which are relatively homogenous, and hpAfrica1 and hpEastAsia [7, 11]. HpAfrica1 can be divided into subtypes hspWAfrica and hspSAfrica (West and South, respectively), while hpEastAsia can be further divided into three subtypes: East Asian (hspEAsia), pacific (hspMaori) and native American (hspAmerind) [7, 12]. Indigenous South American *Helicobacter* isolates from areas of very low population admixture usually belong to the hspAmerind subgroup of hpEastAsia, mirroring the human movement from Asia over Bering Strait and south through the Americas [13]. However, among urban populations of South and Central America, isolates have been shown to be of Western types with different proportions of European and African origin. For example, this has been reported in Colombian [14], Peruvian [13], and Mexican [15] studies, and reflects the exchange of gene pools that occurred in the Americas with the Spanish conquistadores and the African slave trade.

The carcinogenic potential of *H. pylori* has been linked to its virulence factors, mainly the vacuolating cytotoxin *vacA* and the Cag pathogenicity island*, cagPAI* [16, 17]. The *cagPAI* encodes for a type four secretion system (T4SS) together with an effector protein, the cytotoxicity associated virulence factor CagA [18, 19]. CagA is injected into the host cell through the T4SS pili, and initiates a cascade of reactions within the cell. These include dysregulation of cell-cell adhesion and depolarization of the epithelial cell, cellular elongation, increase in IL-8 release, and the activation of NFκB [17]. The EPIYA (glutamic acid-proline- isoleucine-tyrosine-alanine) motifs in the C-terminal region are crucial for the tyrosine phosphorylation of CagA by host kinases [20], and show a variability that has been associated to geographical origin [18, 21]. Most *cagA* positive isolates have the type A and B EPIYA motifs while EPIYA C is characteristic of isolates of European origin, thus termed Western CagA, and EPIYA D is specific to CagA in East Asian isolates and consequently termed East Asian CagA.

The vacuolating cytotoxin (VacA) is present in all *H. pylori and* may induce cytoplasmic vacuoles in eukaryotic cells, form pores in membranes, induce apoptosis, and inhibit T- cells [22]. However, *vacA* shows genetic heterogeneity with differences in activity and *vacA* can also be inactivated by mutations [23]. Two allelic variants have been described in its signal region (s1/s2), in its intermediate region (i1 or i2) and in its middle region (m1/m2) respectively [24, 25]. In these studies it has been shown that the s2 allele is less potent in pore forming and vacuolating capacity while the s1, especially when combined with the i1 allele, is more active.

Adherence of *H. pylori* to the gastric mucosa is highly relevant for the development of gastric disease. The primary modes of *H. pylori* adhesion to the human gastric epithelium are using the blood group antigen-binding adhesin (BabA) that binds to the Lewis b blood group antigen [26, 27] and/or the sialic acid binding adhesin (SabA) that binds to sialyl-Le^x^ and sialyl-Le^a^ [28]. *BabA* has high diversity in the mid and 3’ region suggesting geographic clustering [29]. In addition, the binding affinity to the Leb antigen can vary from strain to strain and can differ up to 1000 fold [30]. Recent investigations have also shown that the Leb binding activity can vary between sequential isolates from the same individuals, suggesting an adaptation of adherence properties [31].

In this study, we aimed to characterize the genomes of *H. pylori* isolated in Nicaragua, which is an area of high gastric cancer risk. We report fifty-two new whole-genome sequenced (WGS) *H. pylori* isolates, collected from patients at different stages of *H. pylori*-associated disease. Of these, 19 pairs of isolates were from within the same individuals enabling within-host comparisons. The isolates were sequenced on the Illumina platform and the genomes were assembled and subsequently annotated and analysed for genomic structure, content, phylogenetic characteristics and virulence gene profiles. Phylogenetic analysis revealed the Nicaraguan *H. pylori* isolates to be most similar to the HspWestAfrica cluster indicating an influence of African ancestry, as well as with urban South- and Central American and European strains, showing no indigenous Amerindian relationship on whole-genome level. The virulence factors *cagA*, *vacA*, *babA* and *sabA* were analysed in more detail and we found that a majority of the isolates carried *cagA*, that the *vacA* s1/i1/m1 genotype co-occurred with *cagA* presence in almost all cases, and that CagA was of western type. Conversely, the adherence factor *babA* was clustering in a distinct Latin American cluster together with both Amerind and urban South- and Central American alleles, which was not seen for e.g., *sabA* indicating different selection pressures on the different adhesion factors.

## Methods

### Patient recruitment

Patients were recruited among individuals undergoing endoscopy due to dyspepsia at Hospital Escuela Antonio Lenin Fonseca (HEALF), Managua, Nicaragua between June and September 2010. Exclusion factors were lack of informed consent, age < 18, prior gastroscopy < = 1 year, prior gastric cancer, and symptoms completely attributable to reflux prior to gastroscopy. One antral and one corpus biopsy were placed in cysteine-broth containing 20 % glycerol, and immediately frozen for shipment and subsequent *H. pylori* culturing as described below. Biopsy samples were obtained from 149 patients, and from these, 32 patients with variable gastric pathology were selected for sequencing of *H. pylori* isolates from antrum and/or corpus biopsies. Among these 32 patients, the median age was 40.75 years (range 18-66 years), 26 were female, and 6 male (Table 1). Twenty of the subjects were from the city area of the capital of Nicaragua, Managua, and nine were from the outskirts of the city rather than the central parts. Three of the subjects lived in rural areas. The study was approved by the Human Research Ethics Committees at Universidad Nacional Autonóma de Nicaragua, Nicaragua, at University of Gothenburg, Sweden, and at University of Toronto, Canada. All individuals included in the cohort agreed to participate in the study and an informed oral and a written consent was obtained from each patient before participation.

**Table 1:** Strain information

Strain	Patient ID	Site^a^	Platform	Age	Sex
Nic01_A	HEALF24875	A	MiSeq	48	M
Nic01_C	HEALF24875	C	MiSeq		
Nic02_A	HEALF27688	A	MiSeq	66	F
Nic03_A	HEALF02414	A	MiSeq	29	F
Nic03_C	HEALF02414	C	HiScan		
Nic04_A	HEALF29487	A	MiSeq	34	F
Nic04_C	HEALF29487	C	MiSeq		
Nic05_A	HEALF24293	A	MiSeq	27	M
Nic05_C	HEALF24293	C	MiSeq		
Nic06_A	HEALF14993	A	MiSeq	47	F
Nic06_A2	HEALF14993	A	MiSeq		
Nic07_A	HEALF27875	A	MiSeq	27	M
Nic07_C	HEALF27875	C	MiSeq		
Nic08_C2	HEALF25546	C	MiSeq	27	F
Nic08_C	HEALF25546	C	MiSeq		
Nic09_A	HEALF19868	A	MiSeq	53	F
Nic09_C	HEALF19868	C	MiSeq		
Nic10_A	HEALF06010	A	MiSeq	35	F
Nic10_C	HEALF06010	C	MiSeq		
Nic11_A	HEALF19162	A	MiSeq	58	M
Nic11_C	HEALF19162	C	MiSeq		
Nic12_A	HEALF08173	A	MiSeq	32	F
Nic12_C	HEALF08173	C	MiSeq		
Nic13_A	HEALF23466	A	MiSeq	30	F
Nic13_C	HEALF23466	C	MiSeq		
Nic14_A	HEALF16065	A	MiSeq	36	F
Nic14_C	HEALF16065	C	MiSeq		
Nic15_A	HEALF12846	A	MiSeq	44	F
Nic15_C	HEALF12846	C	MiSeq		
Nic16_A	HEALF00138	A	MiSeq	24	F
Nic16_C	HEALF00138	C	MiSeq		
Nic17_A	HEALF01245	A	MiSeq	35	F
Nic17_C	HEALF01245	C	MiSeq		
Nic18_A	HEALF08149	A	MiSeq	27	F
Nic18_C	HEALF08149	C	MiSeq		
Nic19_A	HEALF23215	A	MiSeq	47	M
Nic19_C	HEALF23215	C	MiSeq		
Nic20_A	HEALF19582	A	MiSeq	47	F
Nic20_C	HEALF19582	C	MiSeq		
Nic21_A	HEALF02475	A	MiSeq	53	F
Nic21_C	HEALF02475	C	MiSeq		
Pilot, only antrum strains:					
Nic22_A	HEALF02021	A	HiScan	26	F
Nic23_A	HEALF12816	A	HiScan	55	F
Nic24_A	HEALF11221	A	HiScan	56	F
Nic25_A	HEALF04731	A	HiScan	58	M
Nic26_A	HEALF14646	A	HiScan	58	F
Nic27_A	HEALF23077	A	HiScan	40	F
Nic28_A	HEALF10585	A	HiScan	18	F
Nic29_A	HEALF19422	A	HiScan	60	F
Nic30_A	HEALF03699	A	HiScan	53	F
Nic31_A	HEALF15615	A	HiScan	30	F
Nic32_A	HEALF21906	A	HiScan	24	F

Basic information about the patients from which strains were isolated together with sequencing platform and anatomical location of biopsy

^a^Site of biopsy; *A* antrum, *C* corpus

### Collection and culturing of *Helicobacter pylori* isolates

Gastric biopsies were taken during endoscopy in Nicaragua, placed in cysteine-glycerol broth transport media, snap frozen and shipped to Houston, US, on dry ice. The frozen biopsies were then thawed, extracted from the transport tube, and then ground to homogeneity between two sterile slides (frosted end). The homogenized tissues from each biopsy were inoculated onto one non-selective horse blood agar plate (HBA) and one *H. pylori* selective HBA plate. The plates were incubated for 72 hours in a 12 % CO_2_ incubator at 37 °C. The plates were then read and the results were recorded. Negative plates were reincubated, then read every 24 hours up to 14 days. Positive growth was transferred to a fresh HBA plate, and incubated for 48-72 hours. After replating, streaking as per a Kirby-Bauer sensitivity test, and subculturing the organisms for 2-3 days, the plate was gently scraped using a sterile inoculating loop. The loopful of cultured growth was then resuspended in Cysteine Medium and frozen and stored at −70 °C.

The *H. pylori* isolates, as listed in Table 1, were thawed and grown for 48 h at 37 °C under micro aerobic [32] conditions on Columbia agar supplemented with 1 % IsoVitaleX. To obtain a high proportion of *H. pylori*, isolates were recultured for another 48 h. Bacteria were pelleted and frozen in −20 °C pending DNA extraction.

### DNA extraction and cDNA library preparation

Genomic DNA was extracted from *H. pylori*, using the Wizard genomic DNA purification kit (Promega, WI, USA). Pellets from frozen *H. pylori* isolates were hydrated in PBS and subjected to the extraction protocol as described by the manufacturer. DNA contents were measured using the NanoDrop-1000 Spectrophotometer (Thermo Fisher Scientific). All DNA library preparation was performed using TruSeq DNA sample prep v2 Low Throughput (LT) Protocol (Illumina, San Diego, CA) with the gel-based approach selecting for a specified fragment length. Library quality and concentration was measured with Bioanalyzer 2100 (Agilent Technologies, USA) and Qubit 2.0 Fluorometer (Invitrogen, Carlsbad, CA, USA) respectively. The libraries were sequenced at Genomics Core Facility, University of Gothenburg (http://www.genomics.cf.gu.se). The first 12 genomes were sequenced using the HiScanSQ platform (Illumina) paired-end 2 × 100 bp with a mean fragment length of 400 bp. The following 40 genomes were sequenced on the MiSeq platform, paired-end 2 × 250 bp with a fragment mean of 700 bp. All sequencing data is publically available in the Sequence Read Archive database (http://www.ncbi.nlm.nih.gov/sra) under accession number SRP045449.

### Basic data analysis and genome assembly

Fastq files were assessed for sequencing quality using the FastQC software v0.10.1 (www.bioinformatics.babraham.ac.uk/projects/fastqc/) and trimmed with SolexaQA v2.1 Dynamic Trim [33] using the bwa algorithm and a quality score cut-off of 30. The files were subsequently filtered to remove reads < 25 bp as well as reads unpaired after filtering. Prior to assembly, the reads were error corrected using the BayesHammer algorithm in the SPAdes v2.5.1 software [34, 35] and *de novo* assembly was subsequently performed using VelvetOptimiser v2.2.5 (https://github.com/tseemann/VelvetOptimiser) [36]. Assemblies were visualized and compared using ALE and Quast [37]. For detailed assembly statistics, see Additional file 1: Table S1.

### Open reading frame prediction and annotation

For the *de novo* assembled draft genomes, open reading frames (ORFs) were annotated using the Prokka pipeline, v1.9 [38]. This pipeline includes prediction of open reading frames using Prodigal [39], specifically developed for Gram negative bacteria and also rRNA prediction using Barrnap, tRNA prediction using Aragorn, and signal peptide prediction using SignalP. As primary annotation source in Prokka, we used the 26695 genome with the most recent re-annotation [40] and with manually curated outer membrane protein (OMP) annotation according to Alm et al. [41]. Predicted open reading frames with no closely related match in the primary genome was annotated using the global *H. pylori* reference databases, where closely related was defined as having a blastn e-value < 10^−9^. The same pipeline was used to predict ORFs and also to annotate the WGS sequences without ORF/protein information.

### Collection of *H. pylori* genome data and gene annotation

Available complete genomes (*n* = 49) for *H. pylori* were downloaded from public genome database GenBank. We removed the experimental strains B8, Rif1, Rif2, UM298, and UM299 and used the remaining 44 complete strains for comparative genomics, as listed in Additional file 1: Table S2. We also downloaded the whole-genome sequenced isolates available in GenBank as of 2013-11-01, and used all isolates containing open reading frame information but removing strains passaged in animals or experimentally derived strains. A list of the 186 whole-genome shotgun and complete genomes used can be found in Additional file 1: Table S3.

### Comparative genomics

Phylogenetic trees were created in two different ways to reflect different sequence relationships. The whole-genome SNP tree was created using the SNPtree web interface [42]. SNPtree is based on SNP calling with the MUMmer v3 software [43] and FastTree [44] for tree construction. The isolate SouthAfrica7 was used as reference genome, since this isolate is from the most ancient hpAfrica2 phylogeographical group of *H. pylori* [45]. The SNPs were concatenated for each genome and were used to construct a maximum likelihood phylogenetic tree.

Multi-locus sequence analysis (MLSA) is performed by extracting MLST house-keeping gene sequences from draft bacterial genomes. For the MLSA tree we used the nucleotide sequences of the 7 *H. pylori-*specific MLST genes *atpA*, *efp*, *mutY*, *ppa, trpC*, *yphC,* and *ureI* from strain 26695 as reference. The top blastn [46] hit for each of the seven gene sequences were then concatenated for each genome. Multiple sequence alignment was performed using MAFFT v7.221 [47] using the E-INS-i option, suitable for sequences containing conserved motifs embedded between heterogenous regions [48], and a tree was created using the Gubbins software v. 1.4.1 [49]. The phylogenetic trees for all comparisons were constructed and visualized using FigTree v1.4.0 developed by Andrew Rambaut (http://tree.bio.ed.ac.uk/software/figtree/).

### Virulence gene analysis

To pull out the virulence genes from all genomes, a similarity search using blastp was performed on the Nicaraguan genomes and the genomes listed in Additional file 1: Table S2 and Additional file 1: Table S3 collected from GenBank using the BabA (NP_223551.1), SabA (NP_223380.1) and CagA (NP_223213.1) protein sequences from strain J99. Using an e-value cut-off of 10^−9^ the top hit was used for each of the proteins. The hit also had to cover at least 75 % of the length of the query protein to be included in the analysis. These cut-offs resulted in 230 proteins for the CagA comparison, out of which 40 were from Nicaraguan strains, 170 (47 Nicaraguan) proteins being included in the analysis for BabA clustering, and 223 (40 Nicaraguan) proteins being included for SabA. For the alignment analysis of BabA the conserved HPOMP region was trimmed prior to the phylogenetic analysis to allow for the inclusion of shorter sequences. Sequences were aligned using MUSCLE and alignments were manually inspected. PhyML maximum likelihood tree was computed in the SeaView software [50], v4.4.2, which utilize PhyML v. 3.1 and branch support was estimated with the approximate likelihood-ratio test (aLRT), [51] and visualized in FigTree.

*VacA* genotyping was performed by pulling out the vacuolating cytotoxin (*vacA*) genes from each genome the same way as described above, and the allelic variants were determined using primer pairs described previously specific for the signal (s1a/s1b/s2) [24], intermediate (i1/i2) [25], and middle (m1/m2) [24] regions respectively.

To analyse the Nicaraguan *babA* sequences further, the 47 sequences were aligned together with the *babA* sequence of ELS37, an isolate with completed genome sequence from El Salvador (YP_005425388.1) and trimmed down corresponding to the first 500 amino acids of the ELS37 protein. This alignment was first reduced by identity using the ExPASy “Decrease Redundancy” web tool (web.expasy.org/decrease_redundancy/), grouping together sequences sharing 100 % identity to simplify the alignment. To visualize the alignment we used the BOXSHADE software v3.21 (http://www.ch.embnet.org/software/BOX_form.html). To analyse the selection pressure at the different codons of the protein, the 47 Nicaraguan *babA* genomic sequences were obtained using the blastn top hit to the ELS37 *babA* sequence. These were aligned by codon to all full-length *babA* sequences of the complete genomes listed in Additional file 1: Table S2 using MUSCLE and trimmed to the codons corresponding to the first 534 amino acids of the ELS37 BabA protein. Five of the complete sequences not covering the whole length of this alignment were discarded from further analysis. This alignment was then analysed using the Selecton server to identify codons under positive or purifying selection [52] and the results were marked in the BOXSHADE alignment of the Nicaraguan protein sequences. To see if there was any difference in the selection pressure on the global versus Latin American isolates, a subset of the big alignment containing only the Latin American isolates, including the Nicaraguan isolates, were also subjected to Selecton analysis.

To rule out that the variants found in the *babA* sequences were results of *de novo* assembly artefacts we mapped the trimmed sequencing reads back to the *de novo* assembled draft genomes using SMALT (http://www.sanger.ac.uk/science/tools/smalt-0) and manually inspected the coverage over the *babA* region.

## Results

### Nicaraguan isolates are related to hspWestAfrican and urban South- and Central American isolates in whole genome comparison

The genome sequencing of the 52 Nicaraguan isolates from 32 individuals generated on average 295 (131-617) fold coverage and on average 46 contigs per draft genome (for more detailed assembly statistics see Additional file 1: Table S1).

**Fig. 1:**
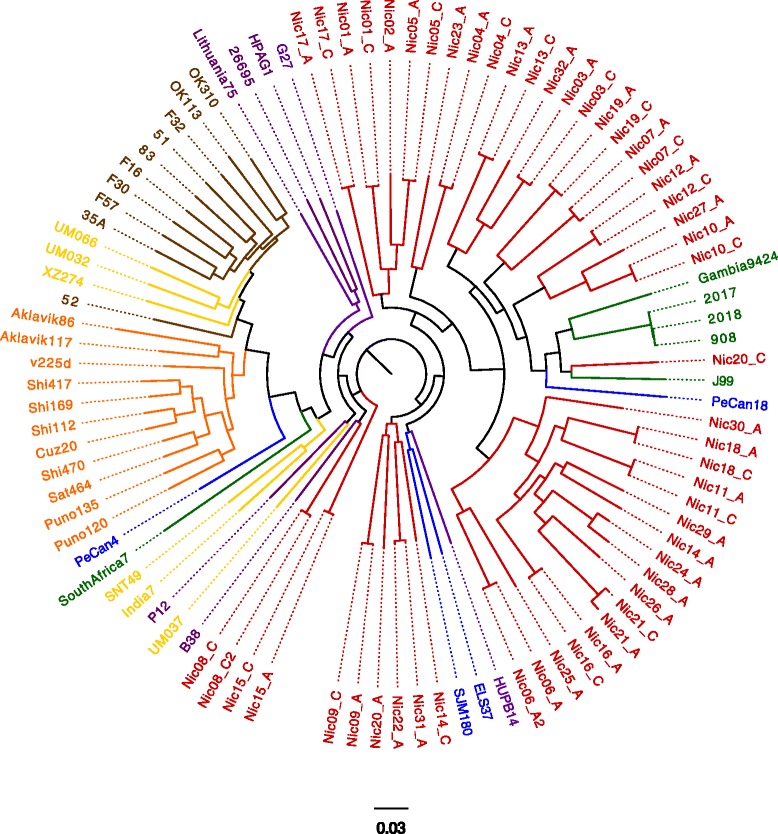
Phylogenetic tree showing the relationship between isolates based on Whole genome (SNP) comparison. Nicaraguan isolates are shown in *red*, urban South- and Central American isolates are shown in *blue*, African isolates in *green* and European isolates in *purple*, East Asian isolates in *brown*, other Asian isolates in *yellow,* and Amerindian isolates in *orange*

Whole genome SNP analysis of the 52 Nicaraguan draft genomes and 44 complete genomes for *H. pylori* isolated worldwide and available in GenBank placed all but four of the Nicaraguan isolates on a separate branch in the tree together with the whole genome sequenced hspWestAfrican isolates J99, Gambia 24/94 and 980, 2017 and 2018, as well as the two urban South- and Central American isolates ELS37 and SJM180 (Fig. 1) and Spanish isolate HUPB14. In addition, the isolates from the corpus (C) and antrum (A) of the same patient clustered closer to each other than isolates from other individuals in all but two cases, the isolates of Nic20 and Nic14 respectively, indicating that specific clones usually establish in hosts but that there are exceptions with co-infection of distantly related isolates. Four isolates from two individuals grouped together with isolates of European and South/Central Asian origin.

### Multi-locus sequence analysis place Nicaraguan isolates closer to urban South American and European isolates

**Fig. 2:**
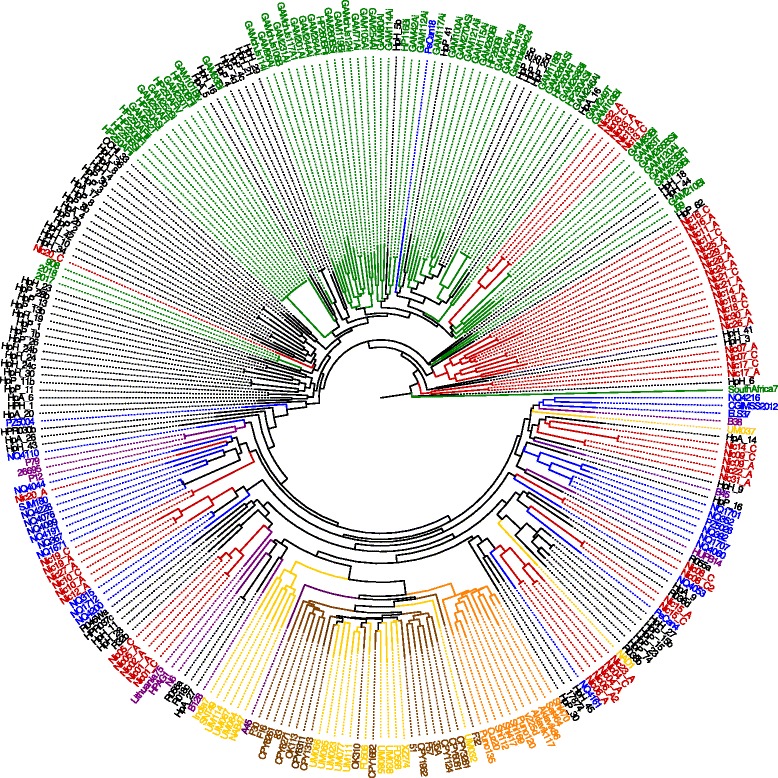
Phylogenetic tree of MLSA similarity, showing the 52 Nicaraguan isolates together with the isolates from the GenBank database. Nicaraguan isolates are shown in *red*, urban South- and Central American isolates are shown in *blue*, African isolates in *green,* North American (the US and Canada) isolates in black, and European isolates in *purple*, East Asian isolates in *brown*, other Asian isolates in *yellow,* and Amerindian isolates in *orange*

To compare the Nicaraguan isolates in a larger context an MLSA phylogenetic tree was constructed using the 44 complete genomes used for SNP phylogeny, all Nicaraguan draft genomes, and available draft genomes deposited in GenBank at the time of analysis (Additional file 1: Table S2 and Additional file 1: Table S3). In total, including the Nicaraguan draft genomes, the comparison comprised of 282 genomes. In this analysis more of the Nicaraguan isolates clustered with urban South- and Central American isolates and European isolates and were more distantly associated with the African isolates than indicated by the SNP tree (Fig. 2). Interestingly, the same isolates that showed a large heterogeneity within one individual in the SNP tree also cluster apart in the MLSA tree.

### Nicaraguan *H. pylori* show high correlation between *cagA* status and *vacA* genotype

**Fig. 3:**
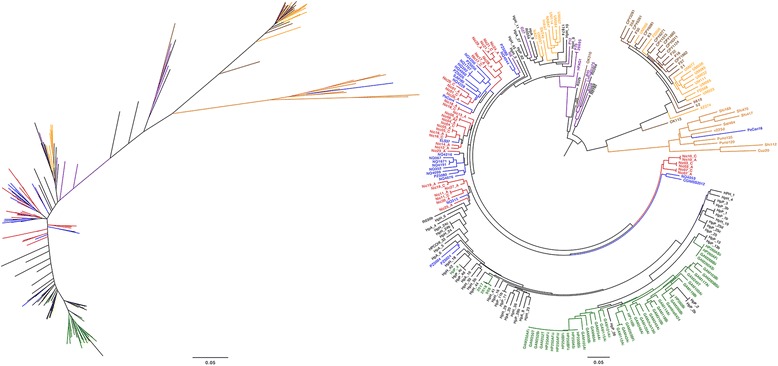
Maximum likelihood (PhyML) tree of CagA protein sequences. Nicaraguan isolates are shown in *red*, urban South- and Central American isolates are shown in *blue*, North American isolates in *black,* African isolates in *green* and European isolates in *purple*, EastAsian isolates in *brown*, other Asian isolates in *yellow* and Amerindian isolates in *orange*

Virulence genes e.g., *cagA* and *vacA* have different alleles that show geographic distribution and also reflects virulence potential [53]. The genomes of the Nicaraguan isolates were screened for the presence of the *cagPAI* and CagA-encoding genes. A total of 40 out of 52 (77 %) were positive for *cagA*. All of the 40 *cagA*-positive isolates showed high similarity to each other along the whole length of the gene and the majority were of EPIYA genotypes A_c_B_c_C but with 10 exceptions carrying double C motifs (ABCC). The CagA profiles of the Nicaraguan isolates studied are hence of the western type. This can also be seen in the maximum likelihood (ML) tree based on CagA protein sequences, in which HpEastAsian isolates group apart from isolates from Indo-Europe, Africa and urban South- and Central America including Nicaragua (Fig. 3). Additionally we could observe that the presence of *cagA* was tightly correlated with the s1/i1/m1 *vacA* genotype where all but two (38/40) of the isolates manifesting this *vacA* genotype were *cagA* positive while none of the isolates (0/10) carrying the s2/i2/m2 combination of alleles also carried *cagA* (Table 2). Two isolates, the corpus and antrum isolate of Nic11 showed an atypical combination of *vacA* alleles carrying the s1 and i1 alleles but a mosaic m portion and *cagA*.

**Table 2:** VacA and CagA genotypes

Strain	*vacA* s allele	*vacA* i allele	*vacA* m allele	CagA EPIYA
Nic01_A	s1	i1	m1	ABCC
Nic01_C	s1	i1	m1	ABCC
Nic02_A	s1	i1	m1	-
Nic03_A	s1	i1	m1	ABC
Nic03_C	s1	i1 nt^a^	m1	ABC
Nic04_A	s1	i1	m1	ABC
Nic04_C	s1	i1	m1	ABC
Nic05_A	s1	i1 nt	m1 nt	ABC
Nic05_C	s1	i1 nt	m1 nt	ABC
Nic06_A	s1	i1 nt	m1	ABC
Nic06_A2	s1	i1 nt	m1	ABC
Nic07_A	s1	i1	m1	ABC
Nic07_C	s1	i1	m1	ABC
Nic08_C2	s2 nt	i2	m2	-
Nic08_C	s2 nt	i2	m2	-
Nic09_A	s2 nt	i2	m2	-
Nic09_C	s2 nt	i2	m2	-
Nic10_A	s1	i1	m1	ABC
Nic10_C	s1	i1	m1	ABC
Nic11_A	s1	i1	m1/m2	ABC
Nic11_C	s1	i1	m1/m2	ABCC
Nic12_A	s1	i1	m1	ABCC
Nic12_C	s1	i1	m1	ABC
Nic13_A	s1	i1 nt	m1	ABCC
Nic13_C	s1	i1 nt	m1	ABCC
**Nic14_A**	**s1**	**i1**	**m1**	**ABC**
**Nic14_C**	**s2**	**i2**	**m2**	**-**
Nic15_A	s2	i2	m2	-
Nic15_C	s2	i2	m2	-
Nic16_A	s1	i1	m1 nt	ABC
Nic16_C	s1	i1	m1 nt	ABC
Nic17_A	s1	i1	m1 nt	ABC
Nic17_C	s1	i1	m1 nt	ABC
Nic18_A	s1	i1	m1	ABCC
Nic18_C	s1	i1	m1	ABCC
Nic19_A	s1	i1	m1	ABC
Nic19_C	s1	i1	m1	ABCC
**Nic20_A**	**s2**	**i2**	**m2**	**-**
**Nic20_C**	**s1**	**i1**	**m1 nt**	**ABC**
Nic21_A	s1	i1	m1	ABC
Nic21_C	s1	i1	m1 nt	ABC
Nic22_A	s2	i2	m2	-
Nic23_A	s1	i1	m1	ABC
Nic24_A	s1	i1	m1 nt	ABC
Nic25_A	s1	i1	m1 nt	ABCC
Nic26_A	s1	i1	m1	ABC
Nic27_A	s1	i1	m1	ABC
Nic28_A	s1	i1	m1 nt	ABC
Nic29_A	s1	i1	m1	ABC
Nic30_A	s1	i1	m1	ABC
Nic31_A	s2	i2	m2	-
Nic32_A	s1	i1	m1	-

Table showing the alleles of the signal (s), intermediate (i), and middle (m) region of the *vacA gene* and the type of C-terminal EPIYA motif of the CagA protein in the different isolates. Isolates in bold show different s/i/m genotype in the antrum and corpus isolate respectively

^a^nt indicating single nucleotide mismatches compared to primer sequence

Interestingly, the two individuals showing a high divergence between the corpus and antrum isolate in whole genome and MLSA comparisons, Nic14 and Nic20, also showed divergent genotypes both for *vacA* allele combination and *cagA* status. In Nic14, the antrum isolate had the s1/i1/m1 *vacA* and carried the *cagA* gene while the corpus isolate was *cagA* negative and had the *vacA* s2/i2/m2 allelic combination. For Nic20 it was the other way around, the antrum isolate being *cagA* negative and *vacA* s2/i2/m2, and the corpus isolate carrier of the more virulent *cagA* and *vacA* s1/i1/m1 combination.

### The outer membrane proteins BabA and SabA show different patterns of geographical clustering: indications of a Latin American branch of BabA

**Fig. 4:**
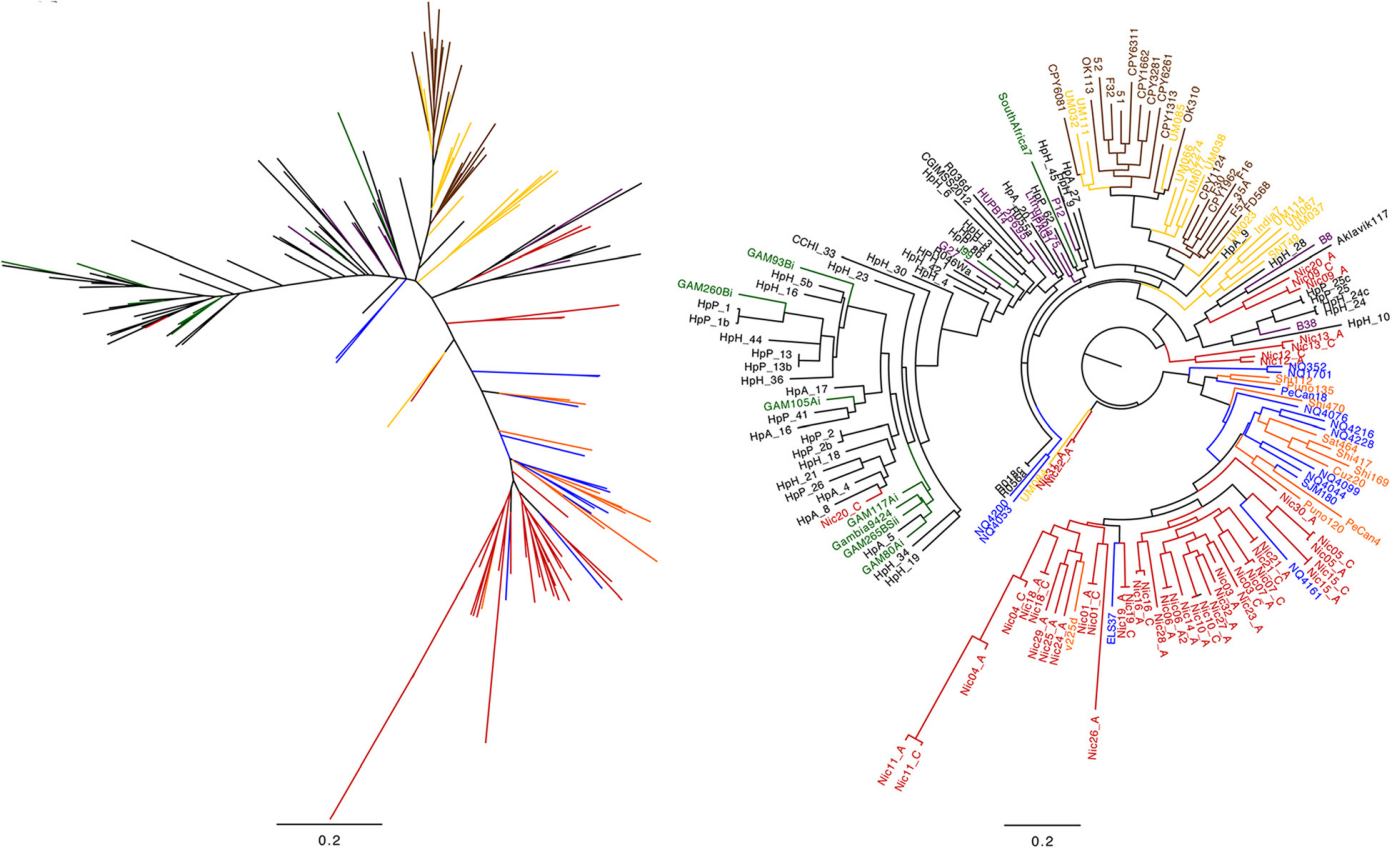
Maximum likelihood (PhyML) tree based on alignment of the 170 BabA protein sequences. Nicaraguan isolates are shown in *red*, urban South- and Central American isolates are shown in *blue*, North American isolates in *black,* African isolates in *green* and European isolates in *purple*, EastAsian isolates in *brown*, other Asian isolates in *yellow* and Amerindian isolates in *orange*

To further analyse virulence gene alleles, we extracted the sequences of the BabA and SabA proteins using blastp [54] and generated phylogenetic trees to compare the genetic distribution. Using the criteria detailed in the Methods section, we extracted BabA protein sequences from 170 isolates, of which 47 were from the Nicaraguan isolates and the rest from the whole-genome sequenced isolates from the database (Additional file 1: Table S2 and Additional file 1: Table S3), which were used to construct a phylogenetic tree (Fig. 4). With the exception of four Nicaraguan isolates, all BabA variants from the Nicaraguan study population clustered with urban South- and Central American isolates and Amerindian isolates forming a Latin American cluster distinctly separated from other alleles retrieved from isolates isolated globally (Fig. 4).

**Fig. 5:**
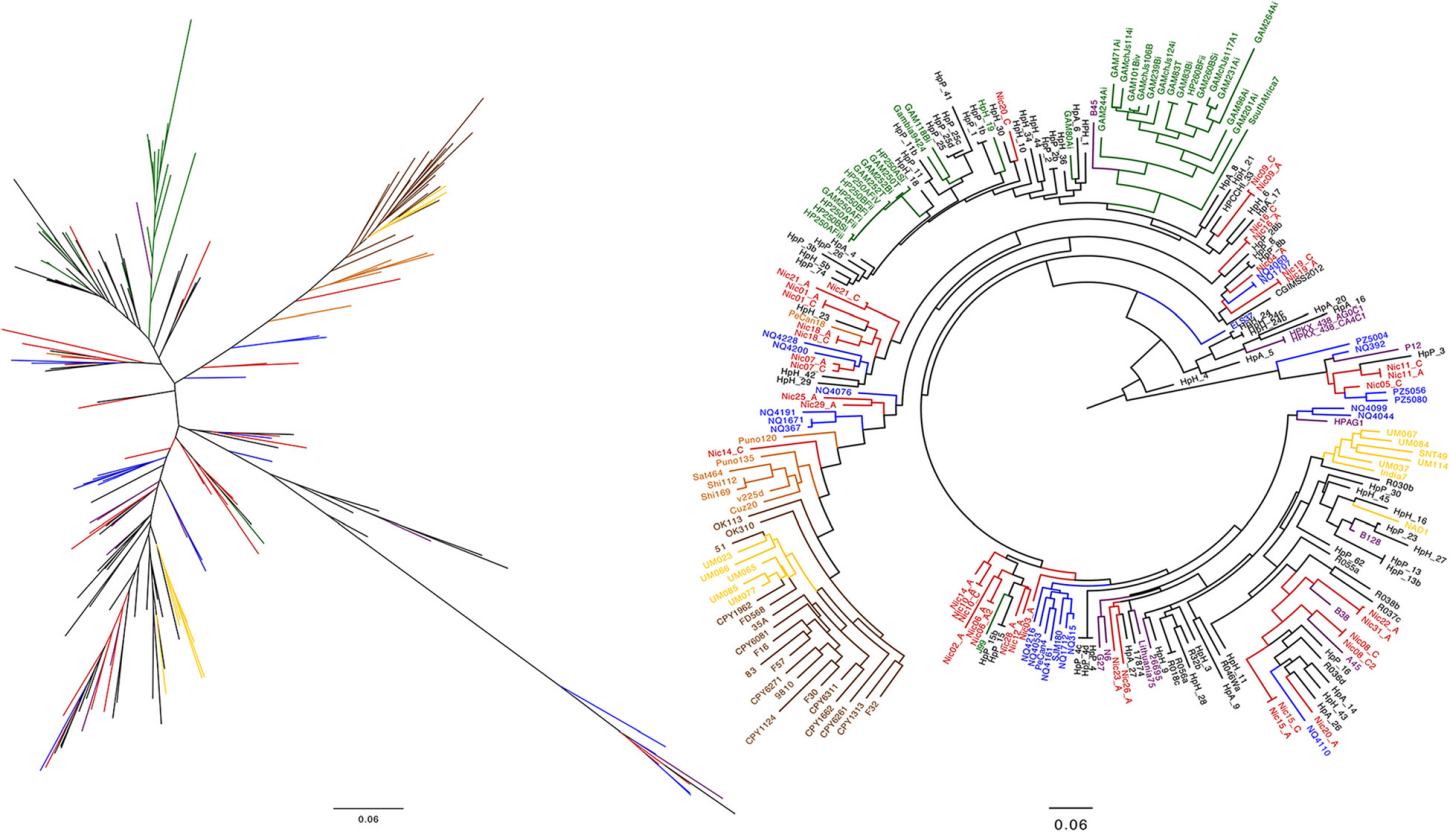
Maximum likelihood (PhyML) based on the alignment of 223 SabA protein sequences. Nicaraguan isolates are shown in *red*, urban South- and Central American isolates are shown in *blue*, North American isolates in *black,* African isolates in *green* and European isolates in *purple*, EastAsian isolates in *brown*, other Asian isolates in *yellow* and Amerindian isolates in *orange*

A similar procedure for SabA generated 223 sequences that were phylogenetically compared (Fig. 5). The SabA protein sequences, contrary to BabA, showed a clustering pattern with respect to geographic location similar to the whole-genome comparisons, with an obvious Asian/Amerind cluster. Comparison of BabA and SabA translated sequences among the isolate pairs from the same individual showed, as noted for the whole-genome comparisons, in most cases more similarity to each other than to isolates from other individuals.

### The Latin American BabA sequences have distinct amino acid positions that are under positive selection

**Fig. 6:**
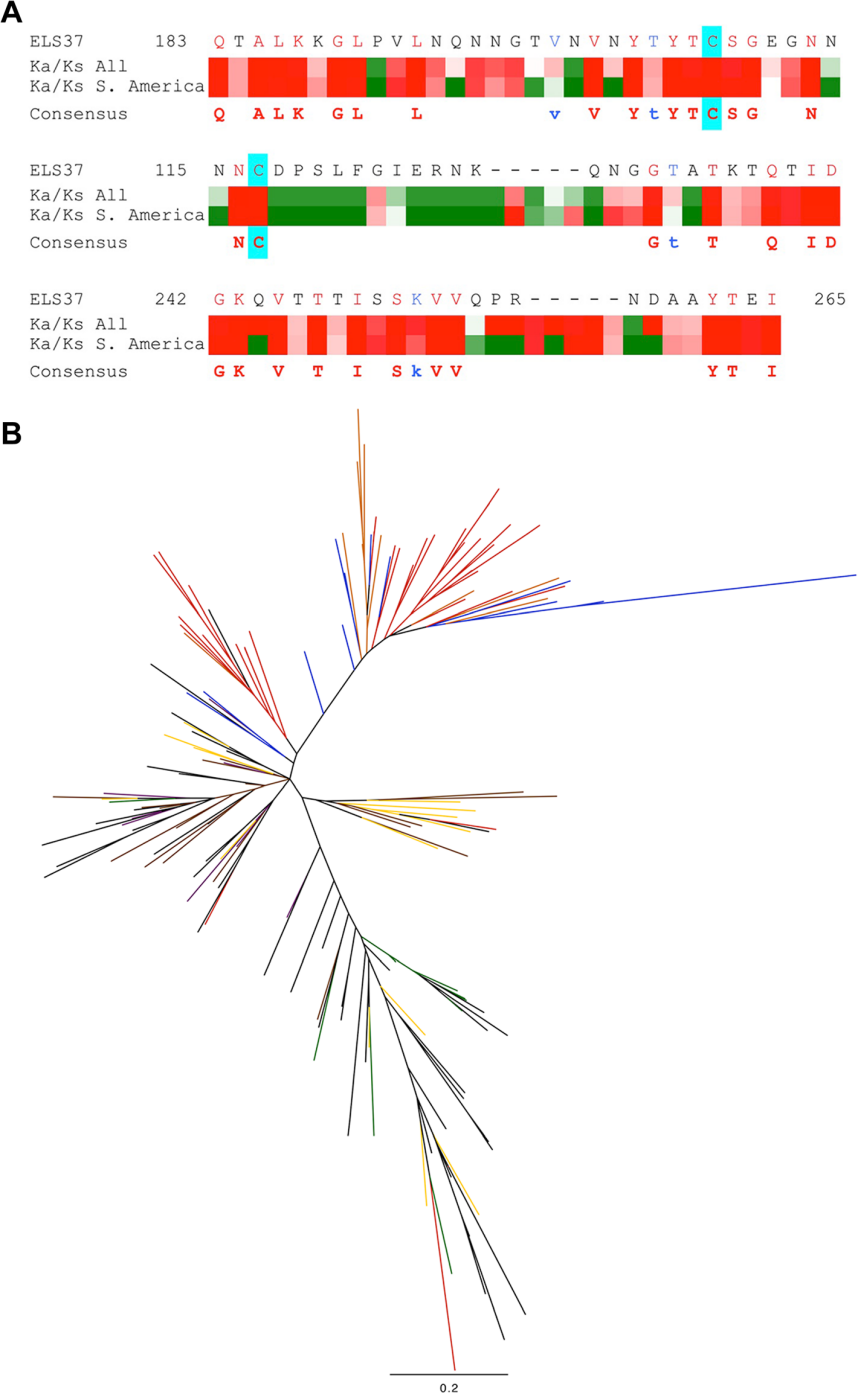
Altered selection pressure in Latin American BabA. **a** Alignment showing Ka/Ks ratio over a region of the BabA protein sequence corresponding to amino acid 183-265 of the ELS37 BabA showing the ELS37 BabA sequence on top and the consensus sequence from the multiple alignment of Nicaraguan BabA proteins at the bottom. Amino acids in red letters are identical in all the isolates and those with blue letters are shared in 90 % of the isolates. Cyan-shaded cysteine residues are predicted to form disulphide bonds constraining the helices of the binding domain inferred from homology to the SabA sequence [62]. Under the ELS37 sequence the Selecton results are shown, the upper line when calculating Ka/Ks based on all complete sequences plus the Nicaraguan sequences and the lower line when basing the Ka/Ks ratio only on the Latin American complete sequences and the Nicaraguan ones. Residues under no selection are white while residues under purifying selection are shown in red and those under positive selection are shown in shades of green. **b** PhyML tree based only on the residues corresponding to those of the alignment in (**a**) of the same 170 BabA protein sequences as in Fig. 4. Nicaraguan isolates are shown in *red*, urban Latin American isolates are shown in *blue*, North American isolates in *black,* African isolates in *green* and European isolates in *purple*, EastAsian isolates in *brown*, other Asian isolates in *yellow* and Amerindian isolates in *orange*

We further determined the characteristics that distinguished the Latin American babA sequences by analysing the babA alleles for signs of selection pressure. Unfortunately, many BabA sequences picked up with blastp were truncated due to contig breaks, usually in the region between amino acids 470 and 570 of the protein. This might be due to that the N-terminal region of BabA is carrying the *H. pylori* outer membrane protein (HPOMP) motif, highly similar between all proteins of the Y-Hop family [41], which poses problems for *de novo* assembly of short read data. The majority of variation in the BabA sequence is however found in the first approximately 500 amino acids, while the rest of the sequence is very conserved with the HPOMP region beginning at approximately 600 amino acids. Due to the high homology and recombination rate between the *babA, babB* and *babC* loci and the above-mentioned assembly problems in these regions we wanted to validate that the babA sequences we analysed had solid support and was not affected by assembly artefacts. We therefore mapped the reads back to the assemblies and manually inspected the alignments over the region. We found that the read coverage was good (>50-fold) in all sequences and that the read pairs spanning the sequences were concordant (results not shown). When extracting the gene sequence of the region corresponding to the first 534 amino acids of the BabA protein from ELS37, an isolate from El Salvador with complete genome sequence, all individuals were displaying isolates with unique *babA* alleles. Also, out of the individuals where we could obtain a *babA* allele from two isolates, 11 showed identical BabA amino acid sequences in the antrum and corpus isolate, while 6 of the isolate pairs showed divergent sequences (Fig. 6). The divergent BabA sequences corresponded to the ancestry determined by WGS, for instance Nic20_C clusters with African isolates (Fig. 1), while Nic20_A clusters in a separate branch with other Nicaraguan and urban South- and Central American isolates. The BabA sequences of Nic20_A and Nic20_C cluster in a similar way in the BabA tree (Fig. 4). Analysis for purifying and positive selection showed that positive selection specific for the Latin American isolates was observed in the middle variable part of BabA (Fig. 6 and Additional file 2: Figure S1).

## Discussion

Nicaragua is a country with high gastric cancer incidence, although no official governmental statistics are available. The incidence of *H. pylori* infection is high and associated with poor living conditions [55]. In this study we sought to determine the genetic variability of clinical isolates isolated from patients with dyspepsia in Nicaragua.

Using whole genome SNP analysis, the majority of the Nicaraguan isolates clustered with West African (Fig. 1) isolates and a few closer to European isolates as has been found in several other studies in Central and South America and most likely reflect the introduction of European and African isolates through the colonization of the Americas and the slave trade from West Africa [15]. We found that 48 isolates clustered more closely to the isolates of African origin while 4 isolates clustered more distant from the other Nicaraguan isolates and showed more resemblance to European isolates. None of the isolates in this study was found within the newly described Amerindian cluster [13, 15] that is related to East Asian isolates and reflects the original indigenous population in the Americas. When using the MLSA with an increased number of isolates for comparison, a larger portion of the Nicaraguan isolates resembled European and urban South- and Central American isolates, as would be expected from isolates isolated from a mestizo population [14]. However, approximately half of the strains still clustered closer to the African isolates. These results match those of other studies in Latin America and also reflect the population structure in Nicaragua. It should however be noted that the use of different analysis methods might change the appearance of phylogenetic trees. Hence, we decided on an approach of using both SNP data and MLSA to analyse the geographical distribution of Nicaraguan *H. pylori* strains.

We found that all *cagPAI*-positive Nicaraguan isolates had the Western type of CagA protein with one or two EPIYA C regions, irrespective of if the phylogenetic analysis showed a more European or African profile (Fig. 3). Interestingly, similar findings in Peru suggest that Latin American *H. pylori* isolates may carry the western CagA type in both HpEurope and hspAmerind isolates. This suggests that the western CagA has spread in Latin American hspAmerind isolates through horizontal transfer and recombination [56]. However, the western type CagA with one C motif was found in 93.9 % of *cagA* positive isolates in Senegal indicating that hpAfrica isolates also carry the western CagA type [53].

For the vacuolating cytotoxin, we could confirm previous observations that the *vacA* s1/m1 allele combination and *cagA* status is tightly linked [22]. Winter and colleagues recently reported, using isogenic, hybrid forms of *vacA*, that the s1/i1 type is the most pathogenic, independently of *cagA* status [57]. They also found that the i1 genotype was strongly associated with the presence of intestinal metaplasia in human subjects without any observed differences in levels of inflammation in the mucosa. The vast majority (83 %) of the Nicaraguan isolates showed the combination of s1 and i1 alleles and all but 2 of these were *cagA* positive, indicating high *H. pylori* virulence in this group of subjects.

The BabA adhesin binds to blood group antigens and is important for *H. pylori* attachment to host cells and to mucins in the stomach. This BabA-mediated binding to cells and mucins has also been shown to facilitate translocation of CagA and upregulate cagA expression, respectively [58, 59]. Studies have suggested that BabA has adapted to the blood group prevalence of the local population. In Europe and US, where blood groups A, B and O are equally common, *H. pylori* isolates expressing BabA binds to all blood groups (generalists) while in populations with predominance of one type, such as the indigenous Amerind population where the majority have type O, a large proportion of the isolates are specialists and only bind to blood group O [30]. However, all Latin American isolates are not specialists [30]. The prevalence of the O blood group in Nicaragua has been reported to be 53-74 % O+ and 0-6 % O- respectively, depending on area [60]. The higher proportions of O can be found in the indigenous population and the lower in the mestizo-dominated urban areas. Interestingly, our results rather indicate that all Latin American isolates, regardless of host genetic background, share a similar variant of BabA. This suggest that Latin American *H. pylori* isolates have adapted to some other, as yet unknown, dietary, environmental or host genetic factor which is specific for Latin America.

The crystal structures of BabA and of the homologous adhesin SabA was recently resolved [61, 62] giving new insights into how *H. pylori* adhesion is mediated on sequence level. Comparing the BabA sequence to the SabA protein structure shows that there are several regions of high positive selection in the region where BabA and SabA do not share homology (Fig. 6). Some of these amino acid positions, especially around the cysteins thought to constrain the binding pocket of these adhesins, show higher positive selection when studying only the Latin American sequences compared to when performing the analysis on all the global strains. This suggests that different evolutionary forces are acting on BabA in isolates colonizing individuals from Latin America than from the rest of the world. The lack of homology between BabA and SabA in these regions implies that this could affect ligand specificity or tropism, which calls for further investigation. A recent study comparing strains from Colombia with strains from the US showed that a higher proportion of the Colombian strains were capable of binding BabA ligand Lewis B (87 % versus 43 % for US strains) [31], also suggesting an association between BabA binding and origin of strain.

The incidence of gastric cancer in Nicaragua is high but no official registry data exist. However, among the in total 149 dyspeptic individuals in this cohort, the pathologist analysis revealed 2 gastric adenocarcinomas (1.4 %) while 18 % of the individuals had extensive (>33 % area) corpus atrophy, and 13 % had intestinal metaplasia in the corpus mucosa (Matteo Fassan, unpublished data), indicating a high incidence of severe pathology in Nicaragua. The finding of Western CagA with C EPIYA motifs fits with the European profile found in the MLSA tree (Fig. 2) but the high proportion of strains carrying a combination of *cagA* and s1/i1 *vacA* allele indicate high virulence in the Nicaraguan strains. Additionally, BabA-mediated binding to the host cells facilitates translocation of CagA [58] and isolates positive for *cagA*, *babA* and *vacA* have been linked to higher oncogenic potential [63]. Although Amerind ancestry is associated with gastric cancer risk, socioeconomic factors and diet play larger roles for cancer development than genetic inheritance in mixed indigenous populations of the Andes [64].

Interestingly, the isolates from two of the individuals (Nic14 and Nic20) showed divergence in both phylogenetic characteristics as well as virulence factor profile, strengthening the hypothesis of variation between clones within the same individual. This could also be observed when it comes to *cagA* and *vacA*, where the two isolates from the same individual in several cases diverged in number of EPIYA C-repeats and, in the two cases mentioned above, also in *cagA* status and *vacA* allele combination (Table 2). This suggests that sequencing only one or few isolates from each individual might be insufficient in providing the full picture of the pathogenic potential of the bacteria within a person.

Our findings of a Latin American phylogenetic group of BabA needs further attention in relation to its presence in cancer patients in Latin America, which was not addressed in this study. Furthermore, gastric cancer is a multifactorial disease and the host responses to the *H. pylori* isolates were also not addressed. The Nicaraguan dietary habits with a high meat and low fruit and vegetable intake as well as high prevalence of smokers in the population most likely also contribute to cancer development. Such parameters, including a more detailed analysis of the circulating *H. pylori* isolates in Nicaragua, needs to be further analysed in patients with gastric cancer in this population.

## Conclusions

We analysed *H. pylori* isolates from patients in Nicaragua and found that they exhibited a whole genome phylogenetic signature of mixed African, urban South- and Central American and European ancestry, reflecting the mixed population in Nicaragua and that none of the isolates were of the Amerind type found in indigenous Latin American people. Interestingly we found that the adhesion factor BabA that mediate adhesion to blood group 0 antigens (Leb) on the stomach epithelium and mucins formed a distinct Latin American phylogenetic group including isolates from the indigenous population. This implies that unknown factors in the stomachs of Latin American individuals selects for a specific type of adhesion properties in *H. pylori*.

## Additional files

Additional file 1:
**Supplementary tables. Table S1.** Assembly statistics. **Table S2.** Complete genomes used for compar ative genomics. **Table S3.** Whole-genome sequenced strains used for comparative genomics. (PDF 210 kb) Additional file 2: Figure S1. Altered selection pressure in South American BabA. Alignment of the Nicaraguan translated BabA sequences corresponding to the first 534 amino acids of the reference, ELS37 BabA. A consensus sequence from the multiple alignment is shown at the bottom where amino acids in red letters are identical in all the isolates and those with blue letters are shared in 90 % of the isolates. The SabA sequence from strain 26695 can be seen aligned at the top and Cyan-shaded cysteine residues are predicted to form disulphide bonds constraining the helices of the binding domain inferred from homology between the BabA and SabA proteins [62]. Similarly, positions shaded in yellow are identical in all SabA and BabA protein sequences (according to Pang et al.) while positions in bold red denotes residues that are identical between SabA and BabA and are predicted to be surface residues in the binding pocket of SabA. Under the ELS37 BabA sequence the Selecton results are shown, the upper line when calculating Ka/Ks based on all complete sequences plus the Nicaraguan sequences and the lower line when basing the Ka/Ks ratio only on the South American complete sequences and the Nicaraguan ones. Residues under no selection are white while residues under purifying selection are shown in red and those under positive selection are shown in shades of green. (PDF 250 kb)
