# Supplementary material for: Identification of a Latin American-specific BabA adhesin variant through whole genome sequencing of Helicobacter pylori patient isolates from Nicaragua
Source: BMC Evol Biol. 2016 Feb 29;16:53. doi: 10.1186/s12862-016-0619-y (PMC4770546; doi:10.1186/s12862-016-0619-y)
Supplement: Additional file 1: — Supplementary tables. Table S1. Assembly statistics. Table S2. Complete genomes used for compar ative genomics. Table S3. Whole-genome sequenced strains used for comparative genomics. (PDF 210 kb) [file 12862_2016_619_MOESM1_ESM.pdf]

Supplementary table 1, Assembly statistics

Strain	Platform	Genome Size (bp)	N50	Assembly coverage (fold)*	Contig number	Predicted ORFs
Nic01_A	MiSeq	1635022	97236	104	43	1554
Nic01_C	MiSeq	1634715	102833	123	43	1551
Nic02_A	MiSeq	1650600	101936	101	57	1523
Nic03_A	MiSeq	1622608	152273	135	29	1541
Nic03_C	HiScan	1611783	167087	424	33	1529
Nic04_A	MiSeq	1679776	108809	182	44	1593
Nic04_C	MiSeq	1675086	59368	153	72	1620
Nic05_A	MiSeq	1652071	79794	160	46	1591
Nic05_C	MiSeq	1648917	83858	115	41	1587
Nic06_A	MiSeq	1673541	78913	130	40	1588
Nic06_A2	MiSeq	1676080	61683	253	47	1598
Nic07_A	MiSeq	1650700	69918	125	37	1569
Nic07_C	MiSeq	1650594	80063	129	33	1570
Nic08_C2	MiSeq	1574456	52590	277	55	1529
Nic08_C	MiSeq	1574718	62001	303	48	1530
Nic09_A	MiSeq	1632217	54886	292	54	1580
Nic09_C	MiSeq	1630444	75481	207	54	1573
Nic10_A	MiSeq	1662645	79604	455	53	1590
Nic10_C	MiSeq	1660232	93535	159	46	1586
Nic11_A	MiSeq	1651432	80206	304	40	1561
Nic11_C	MiSeq	1654044	47705	495	66	1568
Nic12_A	MiSeq	1696688	51457	437	78	1643
Nic12_C	MiSeq	1687089	55209	499	56	1616
Nic13_A	MiSeq	1666823	62724	292	61	1627
Nic13_C	MiSeq	1662354	34480	438	82	1624
Nic14_A	MiSeq	1669747	69948	447	40	1575
Nic14_C	MiSeq	1643796	53712	340	68	1609
Nic15_A	MiSeq	1638822	112091	390	31	1558
Nic15_C	MiSeq	1636264	145448	480	32	1552
Nic16_A	MiSeq	1653644	131180	481	34	1563
Nic16_C	MiSeq	1649746	130872	351	29	1564
Nic17_A	MiSeq	1687918	71512	650	62	1649
Nic17_C	MiSeq	1687001	71942	439	60	1651
Nic18_A	MiSeq	1659406	125192	555	39	1554
Nic18_C	MiSeq	1656362	184347	572	33	1547
Nic19_A	MiSeq	1653912	128740	512	32	1560
Nic19_C	MiSeq	1650301	154963	534	21	1548
Nic20_A	MiSeq	1601554	109971	599	37	1526
Nic20_C	MiSeq	1654727	85059	436	43	1558
Nic21_A	MiSeq	1648378	110422	526	34	1560
Nic21_C	MiSeq	1707280	73869	497	69	1630
Nic22_A	HiScan	1616285	60855	234	80	1569
Nic23_A	HiScan	1663899	82136	331	41	1578
Nic24_A	HiScan	1646116	82696	439	42	1547
Nic25_A	HiScan	1671081	107491	204	42	1554
Nic26_A	HiScan	1648891	156532	361	30	1581
Nic27_A	HiScan	1644340	84225	254	37	1607
Nic28_A	HiScan	1648102	107835	371	29	1562
Nic29_A	HiScan	1644992	116003	458	35	1596
Nic30_A	HiScan	1667014	138911	456	31	1582
Nic31_A	HiScan	1594226	56940	517	54	1572

Supp. Table 2: Complete genomes used for comparative genomics

Organism	Accession number	n.o contigs[a]	n.o plasmids	Size (Mb)	GC%
Helicobacter pylori 2017	NC_017374.1	c	-	1.55	39.3
Helicobacter pylori 2018	NC_017381.1	c	-	1.56	39.3
Helicobacter pylori 26695	NC_000915.1	c	-	1.67	38.9
Helicobacter pylori 35A	NC_017360.1	c	-	1.57	38.9
Helicobacter pylori 51	NC_017382.1	c	-	1.59	38.8
Helicobacter pylori 52	NC_017354.1	c	-	1.57	38.9
Helicobacter pylori 83	NC_017375.1	c	-	1.62	38.7
Helicobacter pylori 908	NC_017357.1	c	-	1.55	39.3
Helicobacter pylori Aklavik117	NC_019560.1	c	2	1.64	38.7
Helicobacter pylori Aklavik86	NC_019563.1	c	2	1.51	39.3
Helicobacter pylori B38	NC_012973.1	c	-	1.58	39.2
Helicobacter pylori Cuz20	NC_017358.1	c	-	1.64	38.9
Helicobacter pylori ELS37	NC_017063.1	c	1	1.67	38.9
Helicobacter pylori F16	NC_017368.1	c	-	1.58	38.9
Helicobacter pylori F30	NC_017365.1	c	1	1.58	38.8
Helicobacter pylori F32	NC_017366.1	c	1	1.58	38.9
Helicobacter pylori F57	NC_017367.1	c	-	1.61	38.7
Helicobacter pylori G27	NC_011333.1	c	1	1.66	38.9
Helicobacter pylori Gambia94 24	NC_017371.1	c	1	1.71	39.1
Helicobacter pylori HPAG1	NC_008086.1	c	1	1.61	39.1
Helicobacter pylori HUP B14	NC_017733.1	c	1	1.61	39.1
Helicobacter pylori India7	NC_017372.1	c	-	1.68	38.9
Helicobacter pylori J99	NC_000921.1	c	-	1.64	39.2
Helicobacter pylori Lithuania75	NC_017362.1	c	1	1.64	38.8
Helicobacter pylori OK113	NC_020508.1	c	-	1.62	38.7
Helicobacter pylori OK310	NC_020509.1	c	1	1.6	38.8
Helicobacter pylori P12	NC_011498.1	c	1	1.68	38.8
Helicobacter pylori PeCan18	NC_017742.1	c	-	1.66	39
Helicobacter pylori PeCan4	NC_014555.1	c	1	1.64	38.9
Helicobacter pylori Puno120	NC_017378.1	c	1	1.64	38.9
Helicobacter pylori Puno135	NC_017379.1	c	-	1.65	38.8
Helicobacter pylori Sat464	NC_017359.1	c	1	1.57	39.1
Helicobacter pylori Shi112	NC_017741.1	c	-	1.66	38.8
Helicobacter pylori Shi169	NC_017740.1	c	-	1.62	38.9
Helicobacter pylori Shi417	NC_017739.1	c		1.67	38.8
Helicobacter pylori Shi470	NC_010698.2	c	-	1.61	38.9
Helicobacter pylori SJM180	NC_014560.1	c	-	1.66	38.9
Helicobacter pylori SNT49	NC_017376.1	c	1	1.61	39
Helicobacter pylori SouthAfrica7	NC_017361.1	c	1	1.68	38.4
Helicobacter pylori UM032	NC_021215.2	c	-	1.6	38.8
Helicobacter pylori UM037	NC_021217.2	c	-	1.69	38.9
Helicobacter pylori UM066	NC_021218.2	c	-	1.66	38.6
Helicobacter pylori v225d	NC_017355.1	c	1	1.6	39
Helicobacter pylori XZ274	NC_017926.1	c	1	1.66	38.6

[a] c stands for completed genome

Supp. Table 3: whole-genome sequenced strains used for comparative genomics

Organism	Assembly	Size (Mb)	GC%
Helicobacter pylori 98-10	ASM17293v1	1.57	38.8
Helicobacter pylori A45	ASM33383v1	1.64	38.6
Helicobacter pylori B128	ASM17295v1	1.65	38.8
Helicobacter pylori CG-IMSS-2012	CGIMSS2012	1.6	39
Helicobacter pylori CPY1124	ASM27522v1	1.56	38.9
Helicobacter pylori CPY1313	ASM27520v1	1.58	38.8
Helicobacter pylori CPY1962	ASM27518v1	1.56	38.8
Helicobacter pylori CPY3281	ASM27472v1	1.61	38.7
Helicobacter pylori CPY6081	ASM27470v1	1.6	38.6
Helicobacter pylori CPY6261	ASM27468v1	1.61	38.7
Helicobacter pylori CPY6271	ASM27466v1	1.6	38.7
Helicobacter pylori CPY6311	ASM27464v1	1.6	38.7
Helicobacter pylori FD506	FD506	1.62	38.7
Helicobacter pylori FD568	FD568	1.61	38.7
Helicobacter pylori GAM100Ai	ASM31000v1	1.64	39.3
Helicobacter pylori GAM101Biv	ASM34494v1	1.62	39.3
Helicobacter pylori GAM103Bi	ASM34496v1	1.62	39.2
Helicobacter pylori GAM105Ai	ASM34498v1	1.67	39.2
Helicobacter pylori GAM112Ai	ASM34500v1	1.63	39.3
Helicobacter pylori GAM114Ai	ASM34502v1	1.62	39.3
Helicobacter pylori GAM115Ai	ASM34504v1	1.69	39.1
Helicobacter pylori GAM117Ai	ASM45576v1	1.64	39.2
Helicobacter pylori GAM118Bi	ASM34508v1	1.68	39.1
Helicobacter pylori GAM119Bi	ASM34510v1	1.66	39.2
Helicobacter pylori GAM120Ai	ASM34512v1	1.69	39.1
Helicobacter pylori GAM121Aii	ASM34514v1	1.67	39.2
Helicobacter pylori GAM201Ai	ASM34506v1	1.62	39.4
Helicobacter pylori GAM210Bi	ASM34516v1	1.62	39.2
Helicobacter pylori GAM231Ai	ASM34518v1	1.63	39.2
Helicobacter pylori GAM239Bi	ASM34520v1	1.63	39.2
Helicobacter pylori GAM244Ai	ASM34522v1	1.6	39.3
Helicobacter pylori GAM245Ai	ASM34524v1	1.65	39.1
Helicobacter pylori GAM246Ai	ASM34526v1	1.67	39.1
Helicobacter pylori GAM249T	ASM34528v1	1.63	39.3
Helicobacter pylori GAM250AFi	ASM34530v1	1.58	39.4
Helicobacter pylori GAM250T	ASM34532v1	1.58	39.4
Helicobacter pylori GAM252Bi	ASM34534v1	1.58	39.4
Helicobacter pylori GAM252T	ASM34536v1	1.58	39.4
Helicobacter pylori GAM254Ai	ASM34538v1	1.64	39.2
Helicobacter pylori GAM260ASi	ASM34540v1	1.63	39.3
Helicobacter pylori GAM260Bi	ASM34542v1	1.67	39.1
Helicobacter pylori GAM260BSi	ASM34544v1	1.58	39.4
Helicobacter pylori GAM263BFi	ASM34546v1	1.65	39.2
Helicobacter pylori GAM264Ai	ASM34548v1	1.61	39.3
Helicobacter pylori GAM265BSii	ASM34550v1	1.67	39.2
Helicobacter pylori GAM268Bii	ASM34552v1	1.64	39.2
Helicobacter pylori GAM270ASi	ASM34554v1	1.65	39.2

Supp. Table 3 continued: WGS strains used for comparative genomics

Organism	Assembly	Size (Mb)	GC%
Helicobacter pylori GAM42Ai	ASM34556v1	1.62	39.3
Helicobacter pylori GAM71Ai	ASM34558v1	1.61	39.3
Helicobacter pylori GAM80Ai	ASM34560v1	1.64	39.2
Helicobacter pylori GAM83Bi	ASM34562v1	1.61	39.4
Helicobacter pylori GAM83T	ASM34564v1	1.61	39.4
Helicobacter pylori GAM93Bi	ASM34566v1	1.62	39.3
Helicobacter pylori GAM96Ai	ASM34568v1	1.66	39.4
Helicobacter pylori GAMchJs106B	ASM34570v1	1.58	39.5
Helicobacter pylori GAMchJs114i	ASM34681v1	1.62	39.3
Helicobacter pylori GAMchJs117Ai	ASM34683v1	1.62	39.3
Helicobacter pylori GAMchJs124i	ASM34685v1	1.62	39.3
Helicobacter pylori GAMchJs136i	ASM34687v1	1.66	39.2
Helicobacter pylori Hp A-11	ASM35964v1	1.67	38.8
Helicobacter pylori Hp A-14	ASM27400v1	1.6	39
Helicobacter pylori Hp A-16	ASM27516v1	1.64	39.2
Helicobacter pylori Hp A-17	ASM27526v1	1.64	39.2
Helicobacter pylori Hp A-20	ASM27528v1	1.67	39.2
Helicobacter pylori Hp A-26	ASM27402v1	1.62	38.9
Helicobacter pylori Hp A-27	ASM27404v1	1.65	38.8
Helicobacter pylori Hp A-4	ASM27530v1	1.67	39.2
Helicobacter pylori Hp A-5	ASM27504v1	1.64	39.2
Helicobacter pylori Hp A-6	ASM27398v1	1.65	39.2
Helicobacter pylori Hp A-8	ASM27382v1	1.64	39.2
Helicobacter pylori Hp A-9	ASM27506v1	1.72	38.7
Helicobacter pylori Hp H-1	ASM34940v1	1.68	39
Helicobacter pylori Hp H-10	ASM27532v1	1.65	39.2
Helicobacter pylori Hp H-11	ASM27448v1	1.66	38.9
Helicobacter pylori Hp H-16	ASM27524v1	1.71	39
Helicobacter pylori Hp H-18	ASM27450v1	1.76	39
Helicobacter pylori Hp H-19	ASM27412v1	1.63	39.3
Helicobacter pylori Hp H-21	ASM27418v1	1.63	39.2
Helicobacter pylori Hp H-23	ASM27416v1	1.65	39.1
Helicobacter pylori Hp H-24	ASM27508v1	1.67	39.2
Helicobacter pylori Hp H-24b	ASM27558v1	1.67	39.1
Helicobacter pylori Hp H-24c	ASM27556v1	1.67	39.2
Helicobacter pylori Hp H-27	ASM27510v1	1.61	38.9
Helicobacter pylori Hp H-28	ASM27512v1	1.63	39
Helicobacter pylori Hp H-29	ASM27388v1	1.68	39.1
Helicobacter pylori Hp H-3	ASM27406v1	1.71	38.9
Helicobacter pylori Hp H-30	ASM27386v1	1.63	39.4
Helicobacter pylori Hp H-34	ASM27426v1	1.63	39.2
Helicobacter pylori Hp H-36	ASM27384v1	1.68	39
Helicobacter pylori Hp H-4	ASM27414v1	1.67	39.1
Helicobacter pylori Hp H-41	ASM27380v1	1.66	39.2
Helicobacter pylori Hp H-42	ASM27390v1	1.7	39.1
Helicobacter pylori Hp H-43	ASM27396v1	1.61	39.1
Helicobacter pylori Hp H-44	ASM27392v1	1.67	39.1

Supp. Table 3 continued: WGS strains used for comparative genomics

Organism	Assembly	Size (Mb)	GC%
Helicobacter pylori Hp H-45	ASM27394v1	1.66	38.9
Helicobacter pylori Hp H-5b	ASM27436v1	1.71	38.9
Helicobacter pylori Hp H-6	ASM27410v1	1.71	38.9
Helicobacter pylori Hp H-9	ASM27408v1	1.64	38.8
Helicobacter pylori Hp P-1	ASM27478v1	1.67	39.1
Helicobacter pylori Hp P-11	ASM27420v1	1.69	39.1
Helicobacter pylori Hp P-11b	ASM27438v1	1.7	39.1
Helicobacter pylori Hp P-13	ASM27422v1	1.71	39
Helicobacter pylori Hp P-13b	ASM27440v1	1.71	39
Helicobacter pylori Hp P-15	ASM27428v1	1.65	38.9
Helicobacter pylori Hp P-15b	ASM27442v1	1.66	38.9
Helicobacter pylori Hp P-16	ASM27430v1	1.55	39.1
Helicobacter pylori Hp P-1b	ASM27444v1	1.67	39.1
Helicobacter pylori Hp P-2	ASM27480v1	1.69	39
Helicobacter pylori Hp P-23	ASM27432v1	1.64	38.8
Helicobacter pylori Hp P-25	ASM27486v1	1.67	39.2
Helicobacter pylori Hp P-25c	ASM27446v1	1.67	39.1
Helicobacter pylori Hp P-25d	ASM27514v1	1.66	39.2
Helicobacter pylori Hp P-26	ASM27538v1	1.7	39
Helicobacter pylori Hp P-28b	ASM27534v1	1.63	39.3
Helicobacter pylori Hp P-2b	ASM27550v1	1.71	39
Helicobacter pylori Hp P-3	ASM27482v1	1.66	39.2
Helicobacter pylori Hp P-30	ASM27540v1	1.64	38.8
Helicobacter pylori Hp P-3b	ASM27552v1	1.66	39.2
Helicobacter pylori Hp P-4	ASM27484v1	1.7	39.1
Helicobacter pylori Hp P-41	ASM27542v1	1.72	39.1
Helicobacter pylori Hp P-4c	ASM27536v1	1.68	39.1
Helicobacter pylori Hp P-4d	ASM27474v1	1.68	39.1
Helicobacter pylori Hp P-62	ASM27544v1	1.65	39.1
Helicobacter pylori Hp P-74	ASM27434v1	1.62	39
Helicobacter pylori Hp P-8	ASM27424v1	1.62	39.3
Helicobacter pylori Hp P-8b	ASM27476v1	1.63	39.3
Helicobacter pylori HP116Bi	ASM34572v1	1.65	39.2
Helicobacter pylori HP250AFii	ASM34574v1	1.59	39.4
Helicobacter pylori HP250AFiii	ASM34576v1	1.58	39.4
Helicobacter pylori HP250AFiV	ASM34578v1	1.59	39.4
Helicobacter pylori HP250ASi	ASM34580v1	1.58	39.4
Helicobacter pylori HP250BFi	ASM34584v1	1.6	39.3
Helicobacter pylori HP250BFii	ASM34586v1	1.59	39.4
Helicobacter pylori HP250BSi	ASM34592v1	1.58	39.4
Helicobacter pylori HP260AFi	ASM34594v1	1.64	39.3
Helicobacter pylori HP260ASii	ASM34598v1	1.64	39.3
Helicobacter pylori HP260BFii	ASM34600v1	1.58	39.5
Helicobacter pylori HPKX_438_AG0C1	ASM17219v1	1.82	39.5
Helicobacter pylori HPKX_438_CA4C1	ASM17221v1	1.57	39.3
Helicobacter pylori N6	ASM28589v1	1.66	38.7
Helicobacter pylori NAB47	ASM25607v2	1.59	39

Supp. Table 3 continued: WGS strains used for comparative genomics

Organism	Assembly	Size (Mb)	GC%
Helicobacter pylori NAD1	ASM25603v2	1.6	38.7
Helicobacter pylori NQ1671	ASM28523v1	1.63	39.1
Helicobacter pylori NQ1701	ASM28513v1	1.64	38.9
Helicobacter pylori NQ1707	ASM28517v1	1.65	38.9
Helicobacter pylori NQ1712	ASM28509v1	1.57	39.1
Helicobacter pylori NQ315	ASM28507v1	1.6	39
Helicobacter pylori NQ352	ASM28511v1	1.64	38.9
Helicobacter pylori NQ367	ASM28521v1	1.62	39.1
Helicobacter pylori NQ392	ASM28515v1	1.65	38.9
Helicobacter pylori NQ4044	ASM27462v1	1.73	38.7
Helicobacter pylori NQ4053	ASM27460v1	1.65	38.9
Helicobacter pylori NQ4060	ASM28519v1	1.65	38.9
Helicobacter pylori NQ4076	ASM27458v1	1.63	39
Helicobacter pylori NQ4099	ASM27456v1	1.65	39
Helicobacter pylori NQ4110	ASM27454v1	1.6	39.1
Helicobacter pylori NQ4161	ASM27452v1	1.64	39
Helicobacter pylori NQ4191	ASM28525v1	1.63	39
Helicobacter pylori NQ4200	ASM27490v1	1.65	38.9
Helicobacter pylori NQ4216	ASM27494v1	1.66	38.9
Helicobacter pylori NQ4228	ASM27492v1	1.65	39
Helicobacter pylori P79	ASM25882v1	1.62	38.9
Helicobacter pylori PZ5004	HP_PZ5004	1.59	38.7
Helicobacter pylori PZ5024	HP_PZ5024	1.52	38.3
Helicobacter pylori PZ5026	HP_PZ5026	1.62	38.7
Helicobacter pylori PZ5056	HP_PZ5056	1.6	38.7
Helicobacter pylori PZ5080	HP_PZ5080	1.61	38.6
Helicobacter pylori PZ5086	HP_PZ5086	1.56	38.8
Helicobacter pylori R018c	ASM29985v1	1.65	38.9
Helicobacter pylori R030b	ASM29969v1	1.61	39.2
Helicobacter pylori R036d	ASM29971v1	1.64	38.9
Helicobacter pylori R037c	ASM29973v1	1.61	38.8
Helicobacter pylori R038b	ASM29975v1	1.63	39
Helicobacter pylori R046Wa	ASM29977v1	1.59	39.1
Helicobacter pylori R055a	ASM29979v1	1.63	38.9
Helicobacter pylori R056a	ASM29981v1	1.65	38.9
Helicobacter pylori R32b	ASM29983v1	1.58	38.9
Helicobacter pylori UM023	UM023	1.62	38.8
Helicobacter pylori UM038	UM038	1.76	38.4
Helicobacter pylori UM065	UM065	1.59	38.9
Helicobacter pylori UM067	UM067	1.68	39
Helicobacter pylori UM077	UM077	1.62	38.8
Helicobacter pylori UM084	UM084	1.66	39.1
Helicobacter pylori UM085	UM085	1.65	38.7
Helicobacter pylori UM111	UM111	1.66	38.7
Helicobacter pylori UM114	UM114	1.71	38.9
