# Supplementary material for: Identification of a Latin American-specific BabA adhesin variant through whole genome sequencing of Helicobacter pylori patient isolates from Nicaragua
Source: BMC Evol Biol. 2016 Feb 29;16:53. doi: 10.1186/s12862-016-0619-y (PMC4770546; doi:10.1186/s12862-016-0619-y)
Supplement: Additional file 2: Figure S1. — Altered selection pressure in South American BabA. Alignment of the Nicaraguan translated BabA sequences corresponding to the first 534 amino acids of the reference, ELS37 BabA. A consensus sequence from the multiple alignment is shown at the bottom where amino acids in red letters are identical in all the isolates and those with blue letters are shared in 90 % of the isolates. The SabA sequence from strain 26695 can be seen aligned at the top and Cyan-shaded cysteine residues are predicted to form disulphide bonds constraining the helices of the binding domain inferred from homology between the BabA and SabA proteins [62]. Similarly, positions shaded in yellow are identical in all SabA and BabA protein sequences (according to Pang et al.) while positions in bold red denotes residues that are identical between SabA and BabA and are predicted to be surface residues in the binding pocket of SabA. Under the ELS37 BabA sequence the Selecton results are shown, the upper line when calculating Ka/Ks based on all complete sequences plus the Nicaraguan sequences and the lower line when basing the Ka/Ks ratio only on the South American complete sequences and the Nicaraguan ones. Residues under no selection are white while residues under purifying selection are shown in red and those under positive selection are shown in shades of green. (PDF 250 kb) [file 12862_2016_619_MOESM2_ESM.pdf]

```
sabA 26695:    1                     EDNGFFVSAGYQIGEAVQMVKNTGELKNLNEKYEQLSQYLNQVASLKQSIQNANNIELVNSSLNYLKSFTNN
ELS37          1 MKKHILSLALGSLLVSALSAEDDGFYTSVGYQIGEAAQMVTNTKGIQQLSDNYENLSKLLTRYSTLNNFIQLASDPSAINAARENLGASAKN
Ka/Ks All
Ka/Ks S. America
```

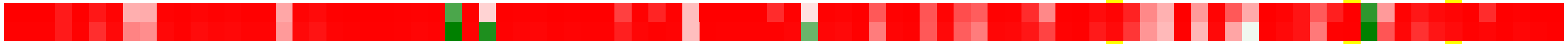

```
Nic03 A        1 MKKHILSLALGSLLVSTLSAEDDGFYTSVGYQIGEAAQMVTNTKGIQQLSDNYENLSKLLTRYSTLNTLIQLSADPSAINAARENLGASAKN
Nic16 A + C    1 MKKHILSLALGSLLVSTLSAEDDGFYTSVGYQIGEAAQMVTNTKGIQQLSDNYENLSKLLTRYSTLNNFIQLASDPSAINVARENLGASAKN
Nic04 C        1 --------------------EDDGFYMSAGYQIGEAAQMVTNTKGIQQLSDNYENLSKLLTRYSTLNTLIQLSADPSAINSARENLGASAKN
Nic09 A        1 MKKHILSLTLGSLLVSTLSAEDDGFYVSAGYQIGEAAQMVKNTKGIQELSDNYEKLNNLLNNYSTLNTLIKLSADPSAINSARENLGASAKN
Nic09 C        1 MKKHILSLTLGSLLVSTLSAEDDGFYVSAGYQIGEAAQMVKNTKGIQELSDNYEKLNNLLNNYSTLNTLIKLSADPSAINSARENLGASAKN
Nic13 C        1 MKKHILSLALGSLLVSTLSAEDDGFYTSVGYQIGEAAQMVSNTKGIQQLSDNYENLSKLLTRYSTLNNFIQLSADPNAINLARENLGASAKN
Nic03 C        1 MKKHILSLALGSLLVSTLSAEDDGFYMSAGYQIGEAAQMVTNTKGIQQLSDNYENLSKLLTRYSTLNNFIQLASDPSAINAARENLGASAKN
Nic30 A        1 MKKHILSLALGSLLVSTLSAEDDGFYMSAGYQIGEAAQMVTNTKGIQQLSDNYENLSKLLTRYSTLNNFIQLASDPSVINAARENLGASAKN
Nic21 A + C    1 MKKHILSLALGSLLVSTLSAEDDGFYTSAGYQIGEAAQMVTNTKGIQQLSDNYENLSKLLTRYSTLNNFIQLASDPSAINAARENLGASAKN
Nic19 A + C    1 MKKHILSLALGSLLVSTLSAEDDGFYTSVGYQIGEAAQMVTNTKGIQQLSDNYENLSKLLTRYSTLNNFIQLASDPSAINAARENLGASAKN
Nic06 A + A    1 MKKHILSLALGSLLVSALSAEDDGFYTSVGYQIGEAAQMVTNTKGIQQLSDNYENLSKLLTRYSTLNNFIQLASDPSAINAARENLGASAKN
Nic15 A + C    1 MKKHILSLTLGSLLVSALSAEDDGFYTSVGYQIGEATQMVKNTKGIQELSDNYENLSKLLTRYSTLNTLIQLSADPSAINSARENLGASAKN
Nic27 A        1 ---HILSLALGSLLVSALSAEDDGFYTSVGYQIGEAAQMVTNTKGIQQLSDNYENLSKLLTRYSTLNNFIQLASDPSVINAARENLGASAKN
Nic18 A + C    1 MKKHILSLALGSLLVSALSAEDDGFYMSAGYQIGEAAQMVTNTKGIQQLSDNYENLSKLLTRYSTLNNFIQLAADPSAINAARENLGASAKN
Nic07 A + C    1 MKKHILSLTLGSLLVSTLSAEDDGFYMSAGYQIGEAAQMVKNTKGIQQLSDNYENLSKLLTRYSTLNNFIQLASDPSVINAARENLGASAKN
Nic12 A        1 MKKHILSLALGSLLVSTLSAEDDGFYTSVGYQIGEAAQMVTNTKGIQQLSDNYENLSKLLTRYSTLNNLIQLSADPSAINAARENLGASAKN
Nic12 C        1 MKKHILSLALGSLLVSTLSAEDDGFYTSVGYQIGEAAQMVTNTKGIQQLSDNYENLSKLLTRYSTLNNLIQLSTDPSAINAARENLGASAKN
Nic10 A + C    1 MKKHILSLALGSLLVSTLSAEDDGFYTSVGYQIGEAAQMVTNTKGIQQLSDNYENLSKLLTRYSTLNNFIQLASDPSAINAARENLGASAKN
Nic32 A        1 MKKHILSLALGSLLVSTLSAEDDGFYTSVGYQIGEAAQMVTNTKGIQQLSDNYENLSKLLTRYSTLNNFIQLASDPSAINAARENLGASAKN
Nic01 A + C    1 MKKHILSLALGSLLVSTLSAEDDGFYTSVGYQIGEAAQMVTNTKGIQQLSDNYENLSKLLTRYSTLNNFIQLASDPSAINAARENLGASAKN
Nic14 A        1 MKKHILSLALGSLLVSTLSAEDDGFYMSAGYQIGEAAQMVKNTKGIQQLSDNYENLSKLLTRYSTLNNFIQLASDPSAINAARENLGASAKN
Nic23 A        1 MKKHILSLALGSLLVSTLSAEDDGFYTSAGYQIGEAAQMVTNTKGIQQLSDNYENLSKLLTRYSTLNTLIQLSADPSAINSARENLGASAKN
Nic28 A        1 MKKHILSLTLGSLLVSTLSAEDDGFYTSVGYQIGEAAQMVTNTKGIQQLSDNYENLSKLLTRYSTLNNLIQLSSDPSAINSARENLGASAKN
Nic25 A        1 MKKHILSLALGSLLVSALSAEDDGFYMSAGYQIGEAAQMVTNTKGIQQLSDNYENLSKLLTRYSTLNTLIQLSADPSAINSARENLGASAKN
Nic05 A + C    1 MKKHILSLALGSLLVSTLSAEDDGFYTSVGYQIGEAAQMVTNTKGIQDLSDRYESLNNLLTRYSTLNTLIKLSADPSAINAARENLGASAKN
Nic29 A        1 MKKHILSLALGSLLVSTLSAEDDGFYMSAGYQIGEAAQMVTNTKGIQQLSDNYENLSKLLTRYSTLNNLIQLSADPSAINSARENLGASAKN
Nic24 A        1 MKKYILSLALGSLLVSTLSAEDDGFYMSAGYQIGEAAQMVTNTKGIQQLSDNYENLSKLLTRYSTLNNFIQLASDPSAINAARENLGASAKN
Nic13 A        1 MKKHILSLALGSLLVSTLSAEDDGFYTSVGYQIGEAAQMVSNTKGIQQLSDNYENLSKLLTRYSTLNNFIQLSADPNAINLARENLGASAKN
Nic26 A        1 MKKHILSLALGSLLVSTLSAEDDGFYTSVGYQIGEAAQMVTNTKGIQDLSDRYESLNNLLTRYSTLNNFIQLASDPSAINAARENLGASAKN
Nic04 A        1 ----------------MLHAEDDGFYTSVGYQIGEAAQMVTNTKGIQQLSDNYENLSKLLTRYSTLNTLIKLSADPSAINAARENLGASAKN
Nic20 A        1 MKKHILSLALGSLLVSTLSAEDDGFYTSVGYQIGEAAQMVKNTKGIQELSDNYEKLNNLLNRYSTLNTLIKLSADPSAINSVRENLGASAKN
Nic20 C        1 MKKHILSLTLGSLLVSTLSAEDDGFYMSAGYQIGEAAQMVKNTKGIQDLSDRYESLNNLLTRYSTLNTLIKLSADPSAINGVRNDLGASAKN
Nic11 A + C    1 MKKHILSLALGSLLVSTLSAEDDGFYTSVGYQIGEAAQMVKNTKGIQELSDNYEKLNNLLNNYSTLNTLIKLSADPSAINSARENLGASAKN
Nic31 A        1 MKKHILSLTLGSLLVSTLSAEDDGFYTSVGYQIGEAAQMVTNTKGIQDLSDNYEKLNNLLTRYSTLNTLIQLSADPSAINSARENLGASAKN
Nic22 A        1 MKKHILSLTLGSLLVSTLSAEDDGFYTSVGYQIGEAAQMVTNTKGIQDLSDNYEKLNNLLTRYSTLNTLIQLSADPSAINSARENLGASAKN
consensus      1 MKK ILSL LGSLLVS L AEDDGFY S GYQIGEA QMV NTKGIQ LSD YE L  LL  YSTLN  I L  DP  IN  R  LGASAKN
```

```
sabA 26695:    72 NYNSTTQSPIFNAVQAVITSVLGFWSLYAGNYFTFFVGKKVG-DSGQOASVQGNPFKTIIENSCS--GIENCAMDQTTYDKMKKLAEDLQAA
ELS37          93 LIGDKANSPAYQAVLLAINAAVGFWNVLGYVTQCGGNGN-QRSTSS-TTVFNNEPGYRSTSITCNYNNLNIGREGVMSIDNMKKINEAYQIL
Ka/Ks All
Ka/Ks S. America
```

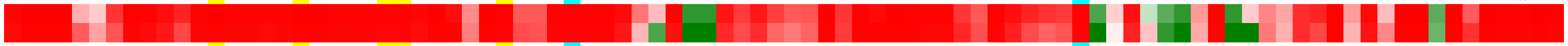

```
Nic03 A        93 LIGDTKNSPAFQAVLLAINAAVGFWNVLGYATQCGGNGD-QKSTSS-TTIFNNEPGYRSTSITCSYNNLGIGREGVMSIDNMKKINEAYQIL
Nic16 A + C    93 LIGDTKNSPAYQAVLLAINAAVGFWNVLGYATQCGGNANGQKSTSS-TTIFNNEPGYRSTSITCDYNNLNIGSQGVMSIENMKKINEAYQIL
Nic04 C        93 LIGDTKNSPAYQAVLLAINAAVGFWNVLGYATQCGGNANGTESTSS-TTIFNNEPGYRSTSITCNYNNLRIGVQGVMSIDNMKKINEAYQIL
Nic09 A        93 LIGDTKNSPAYQAVLLAINAAVGFWNVVGYATQCGGNGN-QKSTSS-TTIFNNEPGYRSTSITCSLNGYAPGYYAPMSIENFKKLNEAYQIL
Nic09 C        93 LIGDTKNSPAYQAVLLAINAAVGFWNVVGYATQCGGNGN-QKSTSS-TTIFNNEPGYRSTSITCSLNGYAPGYYGPMSIENFKKLNEAYQIL
Nic13 C        93 LIGDTKNSPAYQAVLLAINAAVGFWNVVGYVSQCGGNADGQKSTSS-TTIFNNEPGYRSTSITCSLNGYAPGYYGPMSIENFKKLNEAYQIL
Nic03 C        93 LIGDTKNSPAFQAVLLAINAAVGFWNVLGYATQCGGNGD-QKSTSS-TTIFNNEPGYRSTSITCSYNNYAPGVQGPISIKNFKKINEAYQIL
Nic30 A        93 LIGDTKNSPAYQAVLLAINAAVGFWNVLGYATQCGGNGN-QTSTSS-TTIFNNEPGYRSTSIICSYNSLGIGREGVMSIDNMKKLNEAYQIL
Nic21 A + C    93 LIGDTKNSPAYQAVLLAINAAVGFWNVLGYATQCGGNGD-QRSTSS-ITIFNNEPGYRSTSITCSYNNLKIGMEGVMSIDNMKKINEAYQIL
Nic19 A + C    93 LIGDKANSPTYQAVLLAINAAVGFWNVLGYATQCGGNGD-QRSTSS-RTIFNNEPGYRSTSITCSYNNLGIGKEGVMSIDNMKKLNEAYQIL
Nic06 A + A    93 LIGDTKNSPAYQAVLLAINAAVGFWNVLGYATQCGGNGD-QKSTSS-TTIFNNEPGYRSTSITCSYNNLNIGKEAVMSINNMKKINEAYQIL
Nic15 A + C    93 LIGDTKNSPAYQAVLLAINAAVGFWNVLGYATQCGGNGD-QKSTSS-TTIFNNEPGYQSTSITCNYNNLGIGKEGVMSIDNMKKINEAYQIL
Nic27 A        93 LIGDTKNSPAYQAVLLAINAAVGFWNVLGYATQCGGNGD-RRSASS-TTIFNNEPGYRSTSITCNYNNLGIGREGVMSIDNMKKLNEAYQIL
Nic18 A + C    93 LIGDTKNSPAYQAVLLAINAAVGFWNVLGYATQCGGNGD-QKSTSS-TTIFNNEPGYRSTSITCSYNNLGIGREGVMSIDNMKKLNEAYQIL
Nic07 A + C    93 LIGDTKNSPAYQAVLLAINAAVGFWNVLGYATQCGGNGD-QKSTSS-TTIFNNEPGYRSTSITCSYNNLGIGREGVMSIDNMKKLNEAYQIL
Nic12 A        93 LIGDTKTSPAYQAVLLAINAAVGFWNVVGYVTQCGGNGN-QKSTSS-TTIFNNEPGYRSTSITCSLNGLGIGREGVMSIDNMKKINEAYQIL
Nic12 C        93 LIGDTKTSPAYQAVLLAINAAVGFWNVVGYVTQCGGNGN-QKSTSS-TTIFNNEPGYRSTSITCSLNGYSPGYYAPMSIDNFKKLNEAYQIL
Nic10 A + C    93 LIGDTKNSPAYQAVLLAINAAVGFWNVLGYATQCGGNGD-QESTSS-TTIFNNEPGYRSTSITCGYNNLGIGIEGVMSINNMKKINEAYQIL
Nic32 A        93 LIGDTKTSPAYQAVLLAINAAVGFWNVLGYATQCGGNGD-QKSTSS-TTIFNNEPGYRSTSITCSYNNLGIGREGVMSIDNMKKINEAYQIL
Nic01 A + C    93 LIGDTKNSPAYQAVLLAINAAVGFWNVLGYATQCGGNGN-QESTSS-TTIFNNEPGYRSTSIICSYN-LGIGKEAVMSIDNMKKINEAYQIL
Nic14 A        93 LIGDTKNSPAYQAVLLAINAAVGFWNVLGYATQCGGNGD-QKSTSS-TTIFNNEPGYRSTSITCDYNNLGIGSQGVMSIDNMKKLNEAYQIL
Nic23 A        93 LIGDTKNSPAYQAVLLAINAAVGFWNVLGYATQCGGNGD-QQSTSS-TTIFNNEPGYRSTSITCSYNNLGIGREGVMSIDNMKKINEAYQIL
Nic28 A        93 LIGDTKNSPAYQAVLLAINAAVGFWNVLGYATQCGGNGS-QKSTSS-TTVFNNEPGYRSTSITCSYNNLGIGKEGVMSIDNMKKLNEAYQIL
Nic25 A        93 LIGDTKTSPAYQAVLLAINAAVGFWNVLGYATQCGGNGN-KESTSS-TTIFNNEPGYRSTSIICNYNNLGIGREGVMSIDNMKKINEAYQIL
Nic05 A + C    93 LIGDTKNSPAYQAVLLAINAAVGFWNVLGYATQCGGNGD-QKSFSS-TTIFNNEPGYRSTSITCNYNNLGIGREGVMSIDNMKKLNEAYQIL
Nic29 A        93 LIGDTKNSPAYQAVLLAINAAVGFWNVLGYATQCGGNGN-QKSTSS-TTIFNNEPGYRSTSITCGYNNLNIGREGVMSIDNMKKINEAYQIL
Nic24 A        93 LIGDKANSPAYQAVLLAINAAVGFWNVLGYATQCGGNADGTKSTSS-TTIFNNEPGYRSTSITCSSNNLEIGREGVMSIDNMKKINEAYQIL
Nic13 A        93 LIGDTKNSPAYQAVLLAINAAVGFWNVVGYVSQCGGNADGQKSTSS-TTIFNNEPGYRSTSITCSFNRLVTGVQGLISIKNFKKINEAYQIL
Nic26 A        93 LIGDTKNSPAYQAVLLAINAAVGFWNVLGYATQCGGNGN-QQSTSS-MTIFNNEPGYRSTSITCNYNNLGIGREGVMSIDNMKKLNEAYQIL
Nic04 A        93 LIGDTKNSPAYQAVLLAINAAVGFWNVIGYVTQCGGYNKGAQSTPGIQTNFNNEPGYRSTQISCQFGNLRAGVQGPIPIETFKKLNEAYQIL
Nic20 A        93 LIGDTKNSPAYQAVLLAINAAVGFWNVVGYATQCGGNGN-QKSTSS-TTIFNNEPGYRSTSITCSLNRYTPGVQGPMSIDNFKKLNEAYQIL
Nic20 C        93 LIGDKANSPAYQAVLLAINAAVGFWNVVGYVTQCGGNANGTKSTSS-TTIFNNEPGYRSTSITCSLNGYTPGYYGPMSIENFKKLNEAYQIL
Nic11 A + C    93 LIGDTKNSPAYQAVLLAINAAVGFWNIVGYVTQCGGYNKDASSTPGIQTNFNNEPGYRSTQISCQFGNLPAGVQGPMPIETFKKLNEAYQIL
Nic31 A        93 LIGDTKNSPAYQAVLLAINAAVGFWNVVGYVTQCGGNANGTASTSS-TTIFNNEPGYRSTSITCSLNRYTPGYYGPMSIENFKKLNEAYQIL
Nic22 A        93 LIGDTKNSPAYQAVLLAINAAVGFWNVVGYVTQCGGNANGTASTSS-TTIFNNEPGYRSTSITCSLNGYTPGYYGPMSIENFKKLNEAYQIL
consensus      93 LIGD   SP yQAVLLAINAAVGFWNvlGY tQCGG      S     T FNNEPGY ST I C       G  g m I   KKiNEAYQIL
```

```
sabA 26695:  162 QTNSA--------------TKGNNLCALSG---CA--ATDS---TSNPPN-------------
ELS37        183 QTALKKGLPVLNQNNGTVNVNYTYTCSGEGNNNCDPSLFGIERNK-----QNGGTATKTQTIDGKQVTTTISSKVVQPR-----NDAAYTEI
Ka/Ks All
Ka/Ks S. America

Nic03 A      183 QTALKKGLPALKQNDGTINVNYSYTCSGEGNNNCDPSLFEIKNDE-----RSGGTATKTQTIDGKTVTTTISSKVVDWNASGNTSRVSYTEI
Nic16 A + C  184 QTALKKGLPALKQNNGKVDVTYSYTCSGNGNTNCNPSLFGIKGNE-----QNGGSVTKTQTIDGKTVTTTISSKVVDFGASGNTSGVSYTEI
Nic04 C      184 QTALKKGLGALKENNGKIDVTYSYTCSGKGNNNCNPSLFGIKDNE-----RNGGSITTTQTIDGKQVTTKISSKVVDSTASGNTSGVSYTEI
Nic09 A      183 QTALKQGLPALKKSDGTIDVNYSYTCSGEGNNNCDA-----LANK-----RNGGTKTETQTIDGKTVTTTISSKVVDSTASGNTSGVSYTEI
Nic09 C      183 QTALKQGLPALKKSDGTIDVNYSYTCSGEGNNNCDA-----LANN-----RNGGTKTETQTIDGKTVTTTISSKVVDSTASGNTSGVSYTEI
Nic13 C      184 QTALKQGLPALKENNGTINVNYSYTCSGEGNNNCNPSLLGIKDGK-----QNGGTATKTQTIDGKQVTTTISSKVVDSNASGNILHVSYTEI
Nic03 C      183 QTALKNGLPALNNSNGKVNVTYTYTCSGEGNNNCSKEATGVVE-------QNGGTVTKTQTIDGKTVSTTISSKVVDSNASGNTSHVSYTEI
Nic30 A      183 QAALKKGLPALKENNGKVDVNYSYTCSGEGNTNCDPSLFGIKGKG-----RNGGSITKTQIIDGKTVSTTISSKVVDSGASGNTSGVSYTEI
Nic21 A + C  183 QTALKKGLGALNQNNGTVNVDYSYTCSGKGNTNCDPSLFGITGNNENGNGRNGGSITKTQTIDGKSVTTNISSKVVDSTAAGNTTGVSYTEI
Nic19 A + C  183 QTALKKGLPALKENNGTVNVTYTYTCSGEGNTNCDPSLLGIKDEK-----RNGGSTTKTQTIDGKTVTTKINSKVVDSKAQGNTTGVSYTEI
Nic06 A + A  183 QTALKKGLPALKQNNGKVDVSYTYTCSGQGNNNCDPSLFGIKDDK-----RNGGTATKTQTIDGKTVITTISSRVVYSGASGNTSGVSYTEI
Nic15 A + C  183 QAALKKGLPALKQNNGTISVNYSYTCSGEGNNNCNPSLFGINDAQ-----RNGGSITKTQTIDGKQVTTTISSKVVDWNAQGNTSHVSYTEI
Nic27 A      183 QTALKKGLPALKQNDGTINVNYSYTCSGKGNTNCDPSLLGIKDNE-----RNGGTATKTQTIDGKTVTTTISSKVVDSRASGNTMGVSYTEI
Nic18 A + C  183 QTALKKGLGALKQNDGKIDVKYSYTCSGEGNNNCNAVATD----------RNGGTKTETQTIDGKSVTTTISSKVVDSNAQGNTLGVSYTEI
Nic07 A + C  183 QAALKQGLPALNNSNGKVNVTYTYTCSGKGNNNCDPSLFGITSDK-----RNGGTITKTQTIDGKQVSTTISSKVVQPN-----NGAAYTEI
Nic12 A      183 QTALKKGLGALKQNDGTINVNYSYTCSGKGNTNCDPSLFGIKGNTQNGGGRNGGTVTKTQTIDGKTVTTTISSKVVDSRAQGNTLGVSYTEI
Nic12 C      183 QTALKKGLGALKQNDGTINVNYSYTCSGEGNTNCDPSLFGIKGNTQNGGGRNGGTVTKTQTIDGKTVTTTISSKVVDSGAQGNTLGVSYTEI
Nic10 A + C  183 QTALKKGLPALKQNDGTINVNYSYTCSGEGNINCNMSVTGANQ-------QNGGTATKTQTIDGKTVTTRISSRVVDSRASGNTSRVSYTEI
Nic32 A      183 QTALKKGLGALKQNDGTINVSYSYTCSGEGNNNCDPSLLGITNDK-----RNGGTTTKTQTIDGKTVTTTISSKVVQPS-----NGAAYTEI
Nic01 A + C  182 QTALKKGLPALKENNGAIEVKYSYTCSGEGNTNCDPSLFGIEDNK-----RNGGSVTKTQTIDGKQVTTTINSKVVDSGASGNTSRVSYTEI
Nic14 A      183 QTALKKGLPALKQNNGTIDVNYTYTCSGQGNTNCNPSLFGIMGNDQNGHGRNGGSITKPQTIDGKQVSTTISSKVVDSSASGNTSGVSYTEI
Nic23 A      183 QTALKKGLGALKENNGTISVNYSYTCSGERNNNCDPSLFGMKSNE-----RNGGTATKNQTIDGKTVTTTISSKVVDSGEQGNNTGVSYTEI
Nic28 A      183 QTALKKGLGALKENNGTVNVTYTYTCSGEGNTNCDPSLFGMKDEE-----RNSGSITKTQTIDGKTVSTTISSKVVRP------SGAAYTEI
Nic25 A      183 QTALKKGLPALKENNEKVDVNYSYTCSGEGNTNCDKSVTGVTE-------QNGGTKTETQTIDGKQVTTTISSRVVDSSASGNTSGVSYTEI
Nic05 A + C  183 QTALKKGLSALKENNGTINVNYTYTCSGEGNNNCNPSLFGITGNAQNSDRRNGGTATKTQTIDGKSVTTTISSKVVDSGASGNTSGVSYTKI
Nic29 A      183 QTALKKGLGALKQNDGTINVNYSYTCSGERNNNCSQQVTGVDE-------QNGGTTTKTQIIDGKTVTTTISSKVVDSTANGNTSHVSYTEI
Nic24 A      184 QTALKTGLGALKQNNGTISVNYSYTCSGEGNTNCNSGVTGVNK-------QNGGTATKAQTIDGKTVTTTISSKVVDSTAAGNTSGVSYTEI
Nic13 A      184 QTALKQGLPALKENNGTINVNYSYTCSGEGNNNCNPSLLGIKDGK-----QNGGTATKTQTIDGKQVTTTISSKVVDSSASGNTLHVSYTEI
Nic26 A      183 QTALKKGLGALKENNGTINVNYSYTCSGEGNNNCSPLVTGVEN-------QNSGSTTKTQTIDGKQVTTMISSKVVDSNARGNTSHVSYTEI
Nic04 A      185 QTALKKGLGALKENNGKIDVTYSYTCSGEGNNNCNPSLFGMKDDE-----RNGGSTTTTQTIDGKQVTTKISSKVVDSTASGNTSGVSYTEI
Nic20 A      183 QTALKKGLPALKQNNGTIDVNYSYTCSGEGNTNCSPEVTGVNN-------QSNGTTTKTQTIDGKTVTTTISSKVVDSKAQGNTTGVSYTEI
Nic20 C      184 QTALKNGLPALKENNGTVNVTYTYTCSGQGNNNCDALVSDGENK------RNGGSKTETQTIDGKTVITTISSKVVQPS-----NGAAYTEI
Nic11 A + C  185 QTALKKGLPALKENNGTVSVTYTYTCSGEGNNNCK--IEGVE--------QVNGSKTKTQTIDGKEVSTTISSKVVDWNASGNTSGVSYTEI
Nic31 A      184 QTALKKGLPALKENNGTVNVTYSYTCSGEGNTNCNPSLFGINGDR-----RNGGTATKTQTIDGKQVSTTISSKVVDSIASGNTSGVSYTEI
Nic22 A      184 QTALKKGLPALKENNGTVNVTYSYTCSGEGNTNCNPSLFGINGDG-----QNGGTATKTQTIDGKQVSTTISSKVVDSTAPGNTSGVSYTEI
consensus    185 Q ALK GL  L      v V YtYTCSG  N NC                   Gt T  Q IDGK V T I SkVV            YT I
```

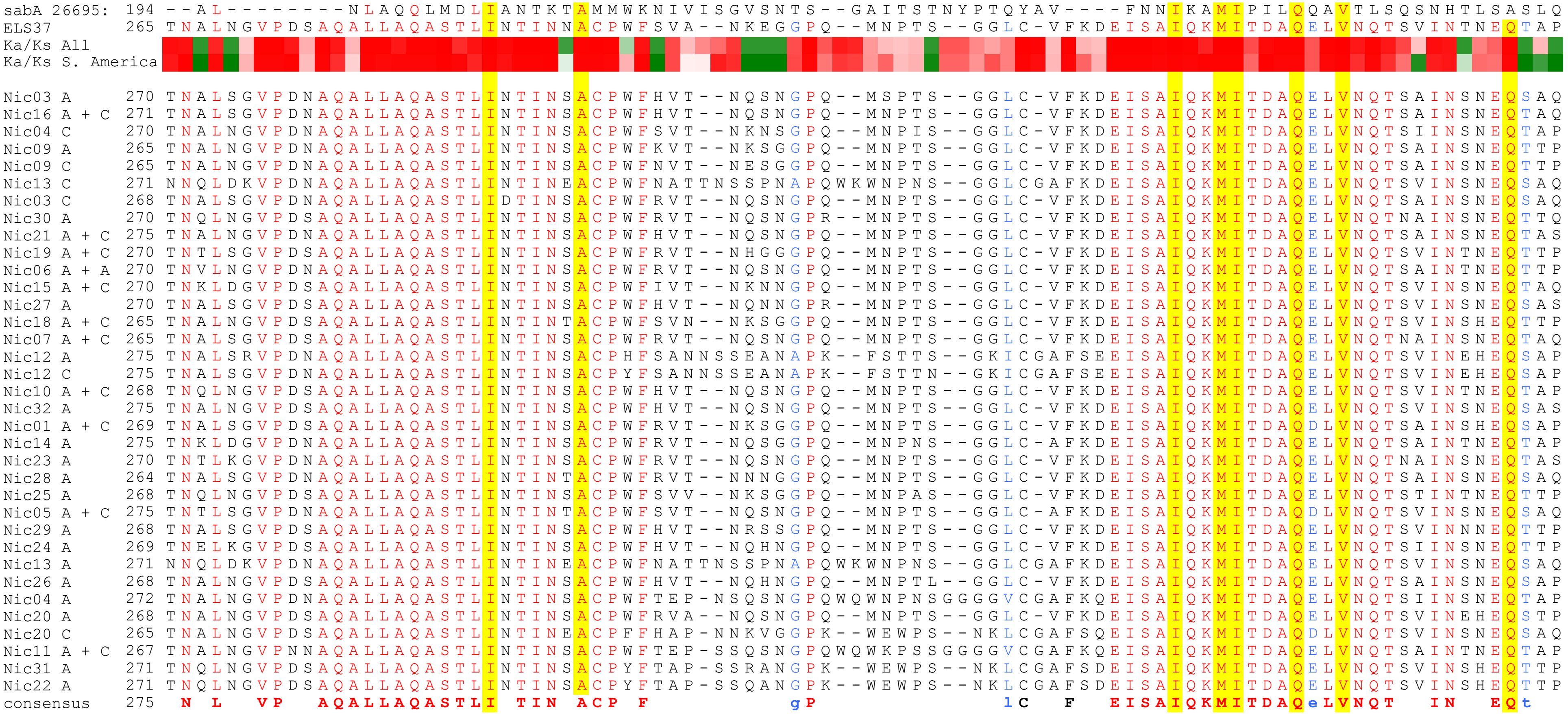

sabA 26695: 194 --AL---------NLAQQLMDLIANTKTAMMWKNIVISGVSNTS--GAITSTNYPTQYAV----FNNIKAMIPILQQAVTLSQSNHTLSASLQ
ELS37 265 TNALNGVPDSAQALLAQASTLINTINNACPWFSVA--NKEGGPQ--MNPTS--GGLC-VFKDEISAIQKMITDAQELVNQTSVINTNEQTAP
Ka/Ks All
Ka/Ks S. America
Nic03 A 270 TNALSGVPDNAQALLAQASTLINTINSACPWFHVT--NQSNGPQ--MSPTS--GGLC-VFKDEISAIQKMITDAQELVNQTSAINSNEQSAQ
Nic16 A + C 271 TNALSGVPDNAQALLAQASTLINTINSACPWFHVT--NQSNGPQ--MNPTS--GGLC-VFKDEISAIQKMITDAQELVNQTSAINSNEQTAQ
Nic04 C 270 TNALNGVPDNAQALLAQASTLINTINSACPWFSVT--NKNSGPQ--MNPIS--GGLC-VFKDEISAIQKMITDAQELVNQTSIINSNEQTAP
Nic09 A 265 TNALNGVPDNAQALLAQASTLINTINSACPWFKVT--NKSGGPQ--MNPTS--GGLC-VFKDEISAIQKMITDAQELVNQTSAINSNEQTTP
Nic09 C 265 TNALNGVPDNAQALLAQASTLINTINSACPWFNVT--NESGGPQ--MNPTS--GGLC-VFKDEISAIQKMITDAQELVNQTSAINSNEQTTP
Nic13 C 271 NNQLDKVPDNAQALLAQASTLINTINEACPWFNATTNSSPNAPQWKWNPNS--GGLCGAFKDEISAIQKMITDAQELVNQTSVINSNEQSAQ
Nic03 C 268 TNALSGVPDNAQALLAQASTLIDTINSACPWFRVT--NQSNGPQ--MNPTS--GGLC-VFKDEISAIQKMITDAQELVNQTSAINSNEQSAQ
Nic30 A 270 TNQLNGVPDSAQALLAQASTLINTINSACPWFRVT--NQSNGPR--MNPTS--GGLC-VFKDEISAIQKMITDAQELVNQTNAINSNEQTTQ
Nic21 A + C 275 TNALNGVPDNAQALLAQASTLINTINSACPWFHVT--NQSNGPQ--MNPTS--GGLC-VFKDEISAIQKMITDAQELVNQTSAINSNEQTAS
Nic19 A + C 270 TNTLSGVPDSAQALLAQASTLINTINSACPWFRVT--NHGGGPQ--MNPTS--GGLC-VFKDEISAIQKMITDAQELVNQTSVINTNEQTTP
Nic06 A + A 270 TNVLNGVPDNAQALLAQASTLINTINSACPWFRVT--NQSNGPQ--MNPTS--GGLC-VFKDEISAIQKMITDAQELVNQTSAINTNEQTTP
Nic15 A + C 270 TNKLDGVPDSAQALLAQASTLINTINSACPWFIVT--NKNNGPQ--MNPTS--GGLC-VFKDEISAIQKMITDAQELVNQTSVINSNEQTAQ
Nic27 A 270 TNALSGVPDSAQALLAQASTLINTINSACPWFHVT--NQNNGPR--MNPTS--GGLC-VFKDEISAIQKMITDAQELVNQTSVINSNEQSAS
Nic18 A + C 265 TNALNGVPDSAQALLAQASTLINTINTACPWFSVN--NKSGGPQ--MNPTS--GGLC-VFKDEISAIQKMITDAQELVNQTSVINSHEQTTP
Nic07 A + C 265 TNALSGVPDSAQALLAQASTLINTINSACPWFRVT--NQSNGPQ--MNPTS--GGLC-VFKDEISAIQKMITDAQELVNQTNAINSNEQTAQ
Nic12 A 275 TNALSRVPDNAQALLAQASTLINTINSACPHFSANNSSEANAPK--FSTTS--GKICGAFSEEISAIQKMITDAQELVNQTSVINEHEQSAP
Nic12 C 275 TNALSGVPDNAQALLAQASTLINTINSACPYFSANNSSEANAPK--FSTTN--GKICGAFSEEISAIQKMITDAQELVNQTSVINEHEQSAP
Nic10 A + C 268 TNQLNGVPDSAQALLAQASTLINTINSACPWFHVT--NQSNGPQ--MNPTS--GGLC-VFKDEISAIQKMITDAQELVNQTSVINTNEQTAP
Nic32 A 275 TNALNGVPDSAQALLAQASTLINTINSACPWFHVT--NQSNGPQ--MNPTS--GGLC-VFKDEISAIQKMITDAQELVNQTSVINSNEQSAS
Nic01 A + C 269 TNALSGVPDSAQALLAQASTLINTINSACPWFRVT--NKSGGPQ--MNPTS--GGLC-VFKDEISAIQKMITDAQDLVNQTSAINSHEQSAP
Nic14 A 275 TNKLDGVPDNAQALLAQASTLINTINSACPWFRVT--NQSGGPQ--MNPNS--GGLC-AFKDEISAIQKMITDAQELVNQTSAINTNEQTAP
Nic23 A 270 TNTLKGVPDNAQALLAQASTLINTINSACPWFRVT--NQSNGPQ--MNPTS--GGLC-VFKDEISAIQKMITDAQELVNQTNAINSNEQTAS
Nic28 A 264 TNALSGVPDNAQALLAQASTLINTINTACPWFRVT--NNNGGPQ--MNPTS--GGLC-VFKDEISAIQKMITDAQELVNQTSAINSNEQSAQ
Nic25 A 268 TNQLNGVPDSAQALLAQASTLINTINSACPWFSVV--NKSGGPQ--MNPAS--GGLC-VFKDEISAIQKMITDAQELVNQTSTINTNEQTTP
Nic05 A + C 275 TNTLSGVPDNAQALLAQASTLINTINTACPWFSVT--NQSNGPQ--MNPTS--GGLC-AFKDEISAIQKMITDAQDLVNQTSVINSNEQSAQ
Nic29 A 268 TNALSGVPDSAQALLAQASTLINTINSACPWFHVT--NRSSGPQ--MNPTS--GGLC-VFKDEISAIQKMITDAQELVNQTSVINNNEQTTP
Nic24 A 269 TNELKGVPDSAQALLAQASTLINTINSACPWFHVT--NQHNGPQ--MNPTS--GGLC-VFKDEISAIQKMITDAQELVNQTSIINSNEQTTP
Nic13 A 271 NNQLDKVPDNAQALLAQASTLINTINEACPWFNATTNSSPNAPQWKWNPNS--GGLCGAFKDEISAIQKMITDAQELVNQTSVINSNEQSAQ
Nic26 A 268 TNALNGVPDSAQALLAQASTLINTINSACPWFHVT--NQHNGPQ--MNPTL--GGLC-VFKDEISAIQKMITDAQELVNQTSAINSNEQSAP
Nic04 A 272 TNALNGVPDNAQALLAQASTLINTINSACPWFTEP-NSQSNGPQWQWNPNSGGGGVCGAFKQEISAIQKMITDAQELVNQTSIINSNEQTAP
Nic20 A 268 TNALNGVPDSAQALLAQASTLINTINSACPWFRVA--NQSNGPQ--MNPTS--GGLC-VFKDEISAIQKMITDAQELVNQTSVINEHEQSTP
Nic20 C 265 TNALNGVPDSAQALLAQASTLINTINEACPFFHAP-NNKVGGPK--WEWPS--NKLCGAFSQEISAIQKMITDAQDLVNQTSVINSNEQSAQ
Nic11 A + C 267 TNALNGVPNNAQALLAQASTLINTINSACPWFTEP-SSQSNGPQWQWKPSSGGGGVCGAFKQEISAIQKMITDAQELVNQTSAINTNEQTAP
Nic31 A 271 TNQLNGVPDSAQALLAQASTLINTINSACPYFTAP-SSRANGPK--WEWPS--NKLCGAFSDEISAIQKMITDAQELVNQTSVINSHEQTTP
Nic22 A 271 TNQLNGVPDSAQALLAQASTLINTINSACPYFTAP-SSQANGPK--WEWPS--NKLCGAFSDEISAIQKMITDAQELVNQTSVINSHEQTTP
consensus 275 N L VP AQALLAQASTLI TIN ACP F gP lC F EISAIQKMITDAQeLVNQT IN EQt

```
sabA 26695:  272 A---TG----SQTNPKFAKDIYTFAQNQKQVISYAQDIFNLFNS-IPAEQYKYLEKAYLKIPNA----------GSTPT----NPYRQ-VVN
ELS37        350 VGSSGGKPFNPFTDASFAQGMLANASAQAKMLDLAHQVGQTLNPNNLSGNFKNFVTGFLATCHNPSTAGTGGTQGSAPGTVTTQTFASGCAY
```

Ka/Ks All
Ka/Ks S. America

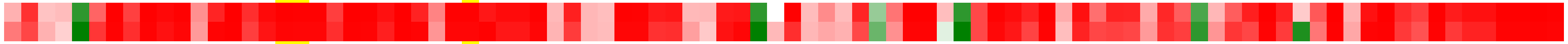

```
Nic03 A      355 VGGNGGKPFNPFTDASFAQGMLANASAQAKMLDLANQVGQTINPSNLSGNFQNFVKNFLATCHNPSTAGTSGTQGSAPGTVTTQTFASGCAY
Nic16 A + C  356 VGGSGGKPFNPFTDASFAQGMLANASAQAKMLNLAHQVGQAINPERLTGTFQNFVKNFLATCHNPSTAGTSGTQGSAPGTVTTQTFASGCAY
Nic04 C      355 VGGSGGKPFNPFTDTSFAQGMLANASAQAKMLDLSHQVGQTLNPDNLSGTFKNFVTGFLATCHNPSTSGANTTQGSAPGTVTTQTFASGCAY
Nic09 A      350 VGGNNGKPFNPYTDASFAQGMLANASAQAKMLDLADQVGQAINPDRLTGTFQNFVKGFLATCNNPSTAGTGGTQGSAPGTVTTQTFASGCAY
Nic09 C      350 VGGNNGKPFNPYTDASFAQGMLANASAQAKMLDLADQVGQAINPDRLTGTFQNFVKGFLATCNNPSTAGTGGTQGSAPGTVTTQTFASGCAY
Nic13 C      361 VGGNNGKPFNPFTDASFAQGMLDNASAQAKMLDLAHQVGQTLNPNNLSGNFQYFVKEYLATCNNPSTAGTGGTQGSAPGTVTTQTFASGCAY
Nic03 C      353 VGGNGGKPFNPFTDASFAQGMLANASAQAKMLDLAHQVGQTINPEKLSGTFQNFVKNFLATCHNPSTAGTGGTQGSAPGTVTTQTFASGCAY
Nic30 A      355 VGGSGGKPFNPFTDASFAQGMLANASAQAKMLNLAHQVGQAINPNNLTGNFKNFVTGFLATCHNPSTAGTGGTQGSAPGTVTTQTFASGCAY
Nic21 A + C  360 VGGSGGKPFNPFTDASFAQGMLANASAQAKMLDLAHQVGQTLNPDNLRETFRSFVKNFLATCHNPSTAGTGGTQGSAPGTVTTQTFASGCAY
Nic19 A + C  355 VGGSGGKPFNPFTDASFAQGMLANASAQAKMLDLAHQVGQTINPNNLSGTFQNFVKNFLATCHNLSTAGTGGTQGSAPGTVTTQTFASGCAY
Nic06 A + A  355 VGGSGGKPFNPFTDASFAQGMLANASAQAKMLDLSHQVGQTLNPSNLSGTFQNFVKNFLATCHNPSTAGTGGTQGSAPGTVTTQTFASGCAY
Nic15 A + C  355 VGGSGGKPFSPFTDASFAQGMLANASAQAKMLDLTHQVGQAIDPSNLSGNFKNFVIGFLATCNNSSTAGTGGTQGSPPGTVTTQTFASGCAY
Nic27 A      355 VGGNGDKPFNPFTDASFAQGMLANASAQAKMLDLSHQVGQTLNPEKLSGTFQDFVKNFLATCHNPSTAGTGGTQGSAPGTVTTQTFASGCAY
Nic18 A + C  350 VGGSGGKPFNPFTDASFAQGMLANASAQAKMLDLAHQVGQTLNPDNLSGTFKNFVTGFLATCHNPSTAGTGGTQGSAPGTVTTQTFASGCAY
Nic07 A + C  350 VGGSGGKPFNPFTDASFAQGMLANASAQAKMLDLSHQVGQTINPNNLSGNFQNFVKNFLATCHNPSTAGTGGTQGSAPGTVTTQTFASGCAY
Nic12 A      363 VGGNNGKPFNPFTDASFAQGMLANASAQAKMLDLAHQVGQTINPSNLSGTFQNFVKNFLATCHNPSTAGTGGTQGSDPGTVTTQTFASGCAY
Nic12 C      363 VGGNNGKPFNPFTDASFAQGMLANASAQAKMLDLAHQVGQTINPSNLSGTFQNFVKNFLATCHNPSTAGTGGTQGSDPGTVTTQTFASGCAY
Nic10 A + C  353 MGGSGGKPFNPFTDASFAQGMLANASAQAKMLDLAHQVGQTLNPINLSGNFQNFVKNFLATCHNPSTAGTGSTQGSAPGTVTTQTFASGCAY
Nic32 A      360 VGGSGGKPFNPFTDASFAQGMLANASAQAKMLDLAHQVGQTLNPSNLNGTFRNFVNNFLATCHNPSTAGTGGTQGSAPGTVTTQTFASGCAY
Nic01 A + C  354 VGNNNGKPFNPFTDASFAQGMLANASAQAKMLDLAHQVGQTLNPDNLSGTFKNFVTGFLATCNNPSTAGTGGTQGSVPGTVTSQTFASGCAY
Nic14 A      360 VGGSGGKPFNPFTDASFAQGMLANASAQAKMLDLAHQVGQTLNPSNLTGTFQNFVKNFLATCHNPSTAGTSGTQGSAPGTVTTQTFASGCAY
Nic23 A      355 VGGSGGKPFNPFTDASFAQGMLANASAQAKMLDLAHQVGQAINPENLSGNFQHFVKNFLATCNNPSTAGTNGTQGSPPGTVTTQTFASGCAY
Nic28 A      349 VGGSGGKPFNPYTDASFAQGMLANASAQAKMLDLVHQVGQAINPERLTGNFQDFVKNFLATCHNPSTAGTGGTQGSAPGTVTTQTFASGCAY
Nic25 A      353 VGGSGGKPFNPFTDASFAQGMLANASAQAKMLNLAHQVGQAINPDNLTRNFKNFVTDFLATCHNPSTAGTSGTQGSAPGTVTTQTFASGCAY
Nic05 A + C  360 VGGNNGKPFNPFTDASFAQGMLANASAQAKMLDLAHQVGQAINPSNLSGNFQSFVKNFLATCHNPSTAGTGGTQGSAPGTVTTQTFASGCAY
Nic29 A      353 VGGNNSKPFNPFTDASFAQGMLANAQAQAKMLNLAEQVGQAINPDRLTGTFKNFVTDFLATCHNPSTAGTSGTQGSAPGTVTTQTFASGCAY
Nic24 A      354 VEGSQGKPFNPFTDASFAQSMLANASAQAKMLDLSHQVGQAINPNNLNRTFQNFVKNFLATCHNPSTAGTSGTQGSAPGTVTTQTFASGCAY
Nic13 A      365 VGGNNGKPFNPFTDASFAQGMLDNASAQAKMLDLAHQVGQTLNPNNLSGNFQYFVKEYLATCNNPSTAGTGGTQGSAPGTVTTQTFASGCAY
Nic26 A      353 VGGNGGKPFNPYTDASFAQSMLANAKAQAEILNQAEQVVK-----NFEKIPKNFVSDSLGVCYE---VQGGERRGTNPGQVTSNTWGAGCAY
Nic04 A      363 VGGSGGKPFNPFTDTSFAQGMLANASAQAKMLDLSHQVGQTLNPDNLSGTFKNFVTGFLATCHNPSTSGANTTQGSAPGTVTTQTFASGCAY
Nic20 A      353 VGTNNGKPFNPFTDASFAQGMLANASAQAKMLDLAHQVGQTINPENLTGNFKNFVTDFLATCNNPSTAGTDGTQGSPPGTVTTQTFASGCAY
Nic20 C      352 VGGN--KPFNPFTDASFAQGMLANAQAQAKMLNLAEQVGQAINPDRLSGTFKNFVTGFLATCNNPSTAGTGGTQGSSPGTVTTQTFASGCAY
Nic11 A + C  358 VGGSGGKPFNPYTDASFAQGMLANASAQAKMLTLANQVGQTINPDNLSGTFKNFVTGFLATCHNPSTAGTGGTQGSPPGTVTTQTFASGCAY
Nic31 A      358 VGGSGGKPFNPFTDASFAQGMLANASAQAKMLDLANQVGQTINPSNLSGTFQNFVKGFLATCNNPY---GNGTQGSPPGTVTTQTFASGCAY
Nic22 A      358 VGGSGGKPFNPFTDASFAQGMLANASAQAKMLDLANQVGQTINPSNLSGTFQNFVKGFLATCNNPY---GNGTQGSPPGTVTTQTFASGCAY
consensus    365 v     KPF PfTD  FA  ML NA AQA mL    QV               FV   La C            Gs PG VTtqTfa GCAY
```

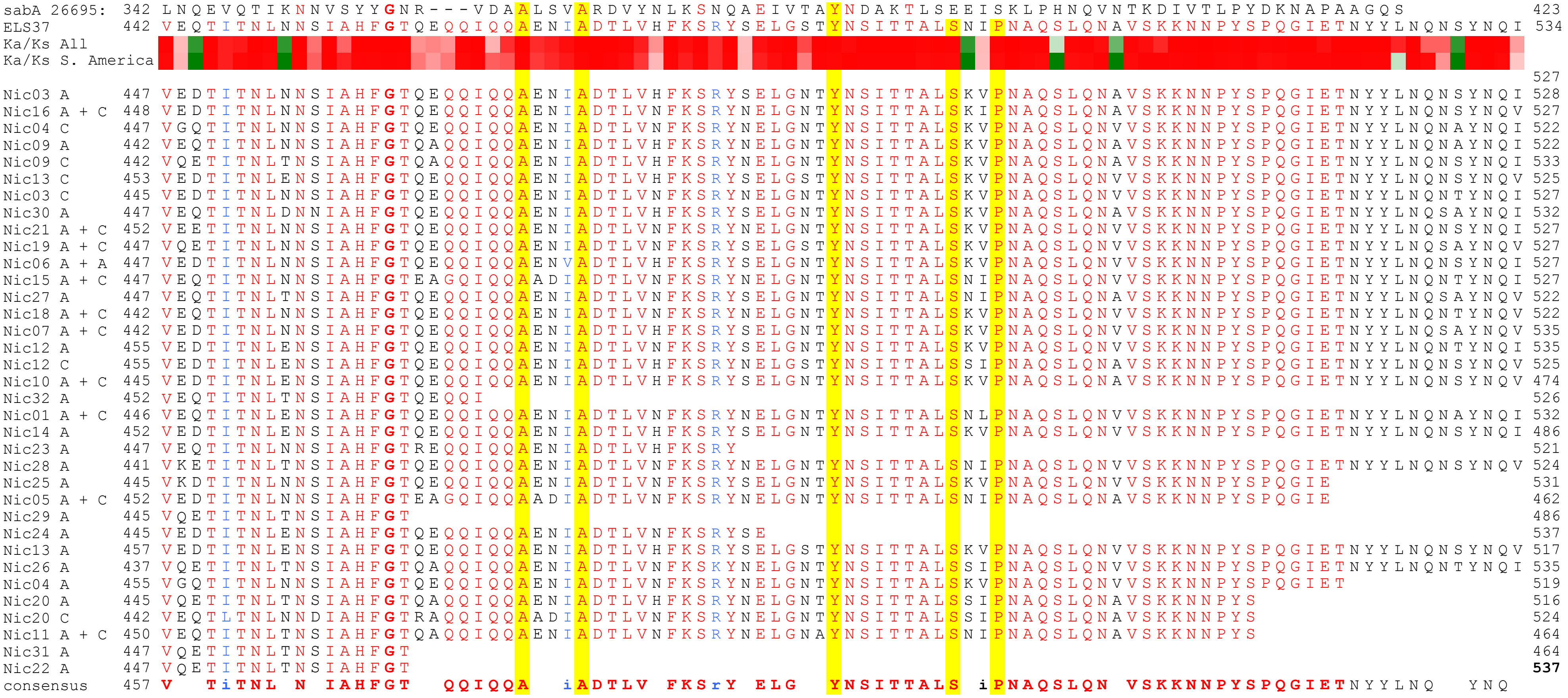

sabA 26695: 342 LNQEVQTIKNNVSYYGNR---VDAALSVARDVYNLKSNQAEIVTAYNDAKTLSEEISKLPHNQVNTKDIVTLPYDKNAPAAGQS 423
ELS37 442 VEQTITNLNNSIAHFGTQEQQIQQAENIADTLVHFKSRYSELGSTYNSITTALSNIPNAQSLQNAVSKKNNPYSPQGIETNYYLNQNSYNQI 534
Ka/Ks All
Ka/Ks S. America
527
Nic03 A 447 VEDTITNLNNSIAHFGTQEQQIQQAENIADTLVHFKSRYSELGNTYNSITTALSKVPNAQSLQNAVSKKNNPYSPQGIETNYYLNQNSYNQI 528
Nic16 A + C 448 VEDTITNLNNSIAHFGTQEQQIQQAENIADTLVNFKSRYNELGNTYNSITTALSKIPNAQSLQNAVSKKNNPYSPQGIETNYYLNQNSYNQV 527
Nic04 C 447 VGQTITNLNNSIAHFGTQEQQIQQAENIADTLVNFKSRYNELGNTYNSITTALSKVPNAQSLQNVVSKKNNPYSPQGIETNYYLNQNAYNQI 522
Nic09 A 442 VEQTITNLNNSIAHFGTQAQQIQQAENIADTLVHFKSRYNELGNTYNSITTALSKVPNAQSLQNAVSKKNNPYSPQGIETNYYLNQNAYNQI 522
Nic09 C 442 VQETITNLTNSIAHFGTQAQQIQQAENIADTLVHFKSRYNELGNTYNSITTALSKVPNAQSLQNAVSKKNNPYSPQGIETNYYLNQNSYNQI 533
Nic13 C 453 VEDTITNLENSIAHFGTQEQQIQQAENIADTLVHFKSRYSELGSTYNSITTALSKVPNAQSLQNVVSKKNNPYSPQGIETNYYLNQNSYNQV 525
Nic03 C 445 VEDTITNLNNSIAHFGTQEQQIQQAENIADTLVNFKSRYNELGNTYNSITTALSKVPNAQSLQNVVSKKNNPYSPQGIETNYYLNQNTYNQI 527
Nic30 A 447 VEQTITNLDNNIAHFGTQEQQIQQAENIADTLVHFKSRYSELGNTYNSITTALSKVPNAQSLQNAVSKKNNPYSPQGIETNYYLNQSAYNQI 532
Nic21 A + C 452 VEETITNLNNSIAHFGTQEQQIQQAENIADTLVNFKSRYNELGNTYNSITTALSKVPNAQSLQNVVSKKNNPYSPQGIETNYYLNQNSYNQI 527
Nic19 A + C 447 VQETITNLNNSIAHFGTQEQQIQQAENIADTLVNFKSRYSELGSTYNSITTALSKVPNAQSLQNVVSKKNNPYSPQGIETNYYLNQSAYNQV 527
Nic06 A + A 447 VEDTITNLNNSIAHFGTQEQQIQQAENVADTLVHFKSRYSELGNTYNSITTALSKVPNAQSLQNVVSKKNNPYSPQGIETNYYLNQNSYNQI 527
Nic15 A + C 447 VEQTITNLNNSIAHFGTEAGQIQQAADIADTLVNFKSRYNELGNTYNSITTALSNIPNAQSLQNVVSKKNNPYSPQGIETNYYLNQNTYNQI 527
Nic27 A 447 VEQTITNLTNSIAHFGTQEQQIQQAENIADTLVNFKSRYSELGNTYNSITTALSNIPNAQSLQNAVSKKNNPYSPQGIETNYYLNQSAYNQV 522
Nic18 A + C 442 VEQTITNLNNSIAHFGTQEQQIQQAENIADTLVNFKSRYNELGNTYNSITTALSKVPNAQSLQNVVSKKNNPYSPQGIETNYYLNQNTYNQV 522
Nic07 A + C 442 VEDTITNLNNSIAHFGTQEQQIQQAENIADTLVHFKSRYSELGNTYNSITTALSKVPNAQSLQNVVSKKNNPYSPQGIETNYYLNQSAYNQV 535
Nic12 A 455 VEDTITNLENSIAHFGTQEQQIQQAENIADTLVNFKSRYSELGNTYNSITTALSKVPNAQSLQNVVSKKNNPYSPQGIETNYYLNQNTYNQI 535
Nic12 C 455 VEDTITNLENSIAHFGTQEQQIQQAENIADTLVHFKSRYNELGSTYNSITTALSSIPNAQSLQNAVSKKNNPYSPQGIETNYYLNQNSYNQV 525
Nic10 A + C 445 VEDTITNLENSIAHFGTQEQQIQQAENIADTLVHFKSRYSELGNTYNSITTALSKVPNAQSLQNAVSKKNNPYSPQGIETNYYLNQNSYNQV 474
Nic32 A 452 VEQTITNLTNSIAHFGTQEQQI 526
Nic01 A + C 446 VEQTITNLENSIAHFGTQEQQIQQAENIADTLVNFKSRYNELGNTYNSITTALSNLPNAQSLQNVVSKKNNPYSPQGIETNYYLNQNAYNQI 532
Nic14 A 452 VEDTITNLENSIAHFGTQEQQIQQAENIADTLVHFKSRYSELGNTYNSITTALSKVPNAQSLQNVVSKKNNPYSPQGIETNYYLNQNSYNQI 486
Nic23 A 447 VEQTITNLNNSIAHFGTREQQIQQAENIADTLVHFKSRY 521
Nic28 A 441 VKETITNLTNSIAHFGTQEQQIQQAENIADTLVNFKSRYNELGNTYNSITTALSNIPNAQSLQNVVSKKNNPYSPQGIETNYYLNQNSYNQV 524
Nic25 A 445 VKDTITNLNNSIAHFGTQEQQIQQAENIADTLVNFKSRYSELGNTYNSITTALSKVPNAQSLQNVVSKKNNPYSPQGIE 531
Nic05 A + C 452 VEDTITNLNNSIAHFGTEAGQIQQAADIADTLVNFKSRYNELGNTYNSITTALSNIPNAQSLQNAVSKKNNPYSPQGIE 462
Nic29 A 445 VQETITNLTNSIAHFGT 486
Nic24 A 445 VEDTITNLENSIAHFGTQEQQIQQAENIADTLVNFKSRYSE 537
Nic13 A 457 VEDTITNLENSIAHFGTQEQQIQQAENIADTLVHFKSRYSELGSTYNSITTALSKVPNAQSLQNVVSKKNNPYSPQGIETNYYLNQNSYNQV 517
Nic26 A 437 VQETITNLTNSIAHFGTQAQQIQQAENIADTLVNFKSKYNELGNTYNSITTALSSIPNAQSLQNVVSKKNNPYSPQGIETNYYLNQNTYNQI 535
Nic04 A 455 VGQTITNLNNSIAHFGTQEQQIQQAENIADTLVNFKSRYNELGNTYNSITTALSKVPNAQSLQNVVSKKNNPYSPQGIET 519
Nic20 A 445 VQETITNLTNSIAHFGTQAQQIQQAENIADTLVHFKSRYNELGNTYNSITTALSSIPNAQSLQNAVSKKNNPYS 516
Nic20 C 442 VEQTLTNLNNDIAHFGTRAQQIQQAADIADTLVNFKSRYNELGNTYNSITTALSSIPNAQSLQNAVSKKNNPYS 524
Nic11 A + C 450 VEQTITNLTNSIAHFGTQAQQIQQAENIADTLVNFKSRYNELGNAYNSITTALSNIPNAQSLQNAVSKKNNPYS 464
Nic31 A 447 VQETITNLTNSIAHFGT 464
Nic22 A 447 VQETITNLTNSIAHFGT 537
consensus 457 V TiTNL N IAHFGT QQIQQA iADTLV FKSrY ELG YNSITTALS iPNAQSLQN VSKKNNPYSPQGIETNYYLNQ YNQ
